# *Neisseria gonorrhoeae* subverts formin-dependent actin polymerization to colonize human macrophages

**DOI:** 10.1371/journal.ppat.1010184

**Published:** 2021-12-28

**Authors:** Stanimir S. Ivanov, Reneau Castore, Maria Dolores Juarez Rodriguez, Magdalena Circu, Ana-Maria Dragoi

**Affiliations:** 1 Department of Microbiology and Immunology, Louisiana State University Health Sciences Center—Shreveport, Shreveport, Louisiana, United States of America; 2 Department of Molecular and Cellular Physiology, Louisiana State University Health Sciences Center—Shreveport, Shreveport, Louisiana, United States of America; 3 Feist-Weiller Cancer Center, Louisiana State University Health Sciences Center—Shreveport, Shreveport, Louisiana, United States of America; Uniformed Services University: Uniformed Services University of the Health Sciences, UNITED STATES

## Abstract

Dynamic reorganization of the actin cytoskeleton dictates plasma membrane morphogenesis and is frequently subverted by bacterial pathogens for entry and colonization of host cells. The human-adapted bacterial pathogen *Neisseria gonorrhoeae* can colonize and replicate when cultured with human macrophages, however the basic understanding of how this process occurs is incomplete. *N*. *gonorrhoeae* is the etiological agent of the sexually transmitted disease gonorrhea and tissue resident macrophages are present in the urogenital mucosa, which is colonized by the bacteria. We uncovered that when gonococci colonize macrophages, they can establish an intracellular or a cell surface-associated niche that support bacterial replication independently. Unlike other intracellular bacterial pathogens, which enter host cells as single bacterium, establish an intracellular niche and then replicate, gonococci invade human macrophages as a colony. Individual diplococci are rapidly phagocytosed by macrophages and transported to lysosomes for degradation. However, we found that surface-associated gonococcal colonies of various sizes can invade macrophages by triggering actin skeleton rearrangement resulting in plasma membrane invaginations that slowly engulf the colony. The resulting intracellular membrane-bound organelle supports robust bacterial replication. The gonococci-occupied vacuoles evaded fusion with the endosomal compartment and were enveloped by a network of actin filaments. We demonstrate that gonococcal colonies invade macrophages via a process mechanistically distinct from phagocytosis that is regulated by the actin nucleating factor FMNL3 and is independent of the Arp2/3 complex. Our work provides insights into the gonococci life-cycle in association with human macrophages and defines key host determinants for macrophage colonization.

## Author summary

During infection, the human-adapted bacterial pathogen *Neisseria gonorrhoeae* and causative agent of gonorrhea can invade the submucosa of the urogenital tract where it encounters tissue-resident innate immune sentinels, such as macrophages and neutrophils. Instead of eliminating gonococci, macrophages support robust bacterial replication. Here, we detail the life cycle of *N*. *gonorrhoeae* in association with macrophages and define key regulators that govern the colonization processes. We uncovered that *N*. *gonorrhoeae* establishes two distinct subcellular niches that support bacterial replication autonomously–one niche was on the macrophage surface and another one was intracellular. Gonococci subverted the host actin cytoskeleton through the actin nucleating factor FMNL3 to invade colonized macrophages and occupy a membrane-bound intracellular organelle. We propose that *N*. *gonorrhoeae* ability to occupy distinct subcellular niches when colonizing macrophages likely confers broad protection against multiple host defense responses.

## Introduction

The betaproteobacteria *Neisseria gonorrhoeae (N*.*g)* is a highly adapted human colonizer and the etiological agent of the sexually transmitted disease gonorrhea. Recently, gonorrhea has emerged once again as a major global public health problem due to increased incidence as well as rapid emergence of antibiotics resistance [1]. A high number of gonococcal infections are asymptomatic, particularly in women, and human-to-human transmission maintains the gonococcal reservoir within the human population [2,3]. Gonococci colonization of the urogenital tract often trigger a localized inflammatory response, which in acute symptomatic infections can progress to the production of purulent exudate that contains gonococci, innate immune cells (macrophages and polymorphonuclear leukocytes- PMNs) and exfoliated epithelial cells [4,5]. A disseminated gonococcal infection can lead to pelvic inflammatory disease, scarring of the fallopian tubes, arthritis and endocarditis [6].

Depending on the exposure route in human infections, gonococci have been found to colonize several mucosa including the genital, ocular, nasopharyngeal and anal [6]. In tissues, gonococci adhere to the host epithelium, proliferate and invade the submucosal region. Interaction of *N*.*g* with epithelial and immune cells, including neutrophils, macrophages, dendritic cells and T cells causes release of inflammatory mediators in animal models and in humans [2,7–9]. Gonococci-encoded immune evasion mechanisms, such as high frequency phase and antigenic variation that diversifies cell surface exposed polypeptides [10,11] and facilitate establishment of persistent infections [2]. Gonococci replication in association with immune phagocytes, including macrophages and neutrophils, as well as resistance to killing has been described [12–21]. Neutrophils recruitment is a central event in gonorrhea progression; however, the role of tissue resident macrophages, comprising ~10% of total leukocytes isolated from the genitourinary mucosa [4], as well as macrophages recruited to sites of acute gonococcal infections [5] remain underexplored. Important work using human cervical tissue explants demonstrated that *N*.*g* colonizes and invades the ectocervical, transformation zone and endocervical region of the female cervix [22], which contain high concentrations of macrophages [23]. Also, macrophages are recruited to the genital tissues [24] and infiltrate the uterine mucosa [25] in murine models of gonorrhea. The mechanism of macrophage colonization by gonococci as well as the identity and subcellular localization of the gonococci-occupied niche remain important open questions.

When associated with different host cells, gonococci have been shown to reside within lysosomes [12,26], in the host cytosol [12,27,28] and on the cell surface [12,28,29]. Gonococci tether to epithelial cells and neutrophils via type IV pili [30–32], which subsequently facilitates the binding of gonococcal adhesins, such as the outer membrane protein PorB [33,34] and the Opacity (Opa) proteins family [35–38], to host surface receptors, such as the human carcinoembryonic antigen-related cell adhesion molecule (CEACAM) receptor family [39]. Gonococci encode up to 11 *opa* loci [40,41] which give rise to 7 to 9 unique Opa proteins with distinct binding affinities for the different CEACAM receptors and interactions with CEACAM1, CEACAM3, CEACAM5 and CEACAM6 have been reported [42–45]. The broadly expressed CEACAM1 receptor mediates attachment to neutrophils and epithelial cells [45], whereas engagement of the neutrophil specific CEACAM3 receptor transports gonococci to a degradative compartment [46–48].

On host cells as well as inanimate surfaces gonococci aggregate to form colonies as a direct result of dynamic interactions mediated by Opa proteins, gonococcal lipooligosaccharirides, and the type IV pili [49–51]. Gonococci and the closely related meningococci colonize the surface of epithelial cell to form colonies where plasma membrane proximal bacteria are embedded within membrane ruffles enriched in polymerized actin microfilaments, also known as actin plaques [52–56] and are proposed to remain epicellular [57]. Retraction of type IV pili emanating from surface-associated gonococcal colonies triggers actin polymerization directly underneath the bacterial colony [53,55,56]. A number of host cell surface receptors and adhesion molecules (EGFR, CD46, CD44 and ICAM) that engage the cortical actin network also aggregate within the gonococci-induced actin plaques [55,58]. Ezrin–an adaptor protein that link surface receptors with the cortical actin cytoskeleton is also enriched within these structures [52,55]. Actin plaques are likely produced as a result of surface receptor clustering by the gonococci type IV pili as formation depends on the cholesterol content of the plasma membrane [59]. Internalized diplococci have been observed in epithelial cells; however, they represent a minor fraction of the total cell-associated bacterial population [55]. On neutrophils, gonococci have been observed on the cell surface as well as intracellularly [27–29,60–62].

Dynamic remodeling of the host actin cytoskeleton by bacterial pathogens is a critical mechanism for host invasion and spreading mediated by both Arp2/3-dependent and formin-dependent mechanisms [63,64]. The Arp2/3 complex initiates branched actin formation by binding to the sides of pre-existing filaments, whereas the formin family of actin nucleators directs the elongation of unbranched actin filaments [65]. Here, we investigate the molecular processes mediating human macrophage colonization by gonococci and define at the molecular level the distinct subcellular niches occupied by the bacteria that support replication. Also, we provide evidence for a novel mechanism regulated by the host formin FMNL3 that allows bacterial colonies rather than individual diplococci to invade and colonize human macrophages intracellularly.

## Results

### N.g colonizes and replicates in association with human macrophages

**Fig 1:**
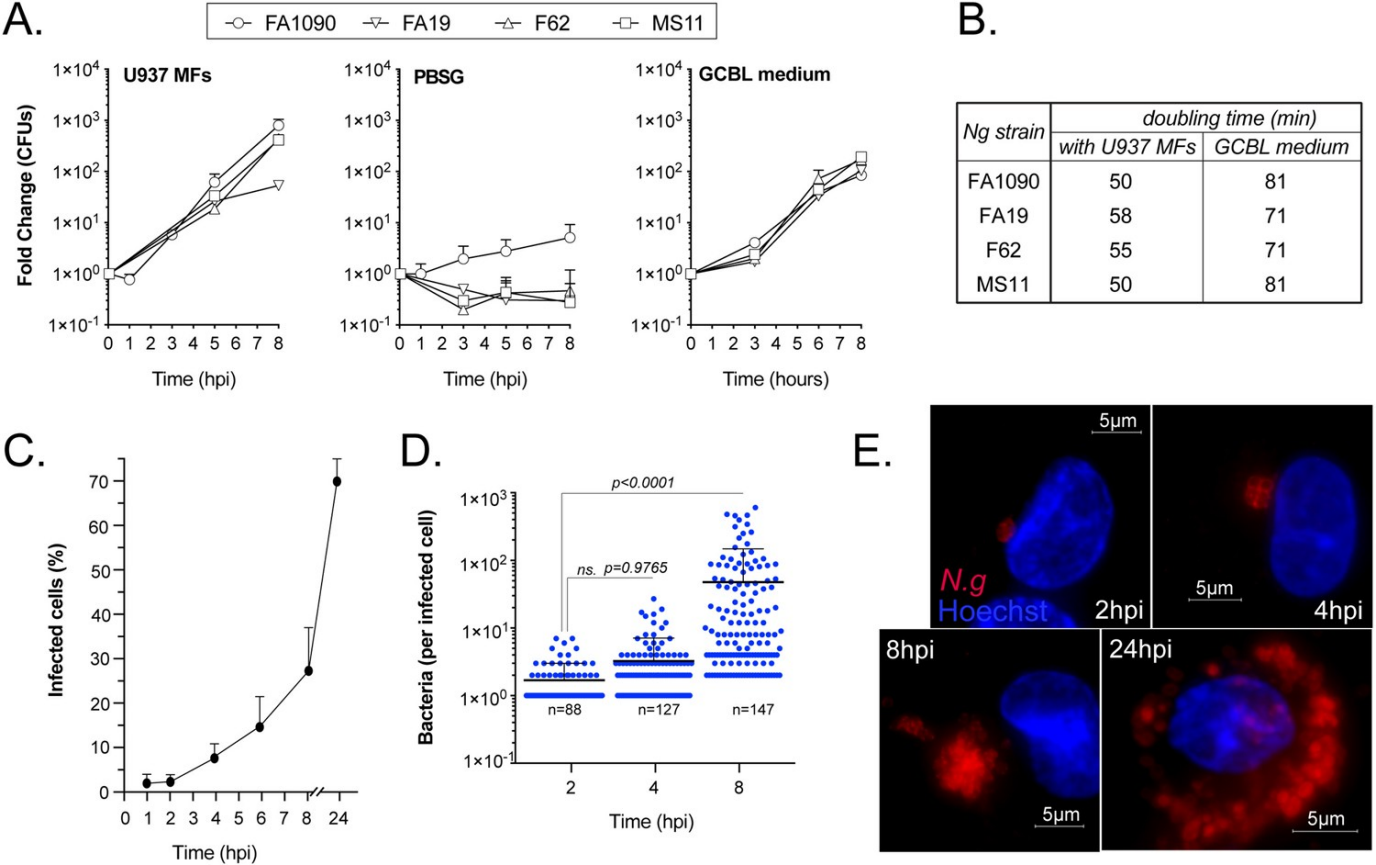
*N*. *gonorrhoeae* associates and replicates with human macrophages. **(A)** Growth of different *N*.*g* strains cultured in the presence/absence of differentiated U937 human macrophages (U937 MFs) at MOI = 0.1 or in axenic rich liquid culture media GCBL. **(B)** Growth rate of gonococcal strains when cultured with U937 MFs or in GCBL media calculated from recovered CFUs counts. **(C-E)** Microscopy analysis of gonococcal growth kinetics of N.g FA1090 with U937 MFs at MOI = 0.1 was performed on images from fixed samples at different time points. The percentage of infected macrophages **(C)** and the number of bacteria per cell **(D)** are shown. **(D)** The number of bacteria in each gonococcal colony were determined by mathematical conversion of 3D microscopy volume measurements. **(E)** Representative micrographs of *N.g* FA1090 infected U937 MFs at the indicated time-points. **(A-E)** Representative experiments from two **(B)** or at least three **(A, C-E)** biological replicates are included. **(A and C)** Mean of technical replicates ± SD are shown. **(D)** Each data point represents a single macrophage; Means ± SD are shown; one-way ANOVA analysis, not significant (ns.).

To analyze the capacity of human macrophages to support gonococcal replication we performed distinct growth assays. First, we quantified the bacterial colony forming units (CFUs) recovered at discrete times post infection in cell culture experiments. These infections we completed under nutrients-depleted conditions in PBSG media (PBS, 7.5mM glucose, 900μM CaCl_2_, 500μM MgCl_2_) because PBSG itself does not support gonococcal replication [12] (Fig 1A). In the presence of human macrophages differentiated from the pro-monocytic U937 cell line, gonococcal CFUs increased by 2 to 3 log_10_ in an 8-hour period (Fig 1A and 1B). Using microscopy, we determined that the percentage of infected U937 MFs increased from 2 to 28% and the average number of gonococci per macrophage also increased from 1.6 to 47.7 in that time frame (Figs 1C–1E and S1). The growth kinetics for strains isolated from patients with disseminated (FA1090, FA19) or uncomplicated (F62, MS11) gonococcal infections [66] in U937 macrophages (U937 MFs) infections were similar (Fig 1A and 1B). Taken together, these data from cell culture infections with a low starting multiplicity of infection (MOI) demonstrate the gonococci capacity to colonize human macrophages, replicate and potentially disseminate to neighboring bystander cells within hours.

**Fig 2:**
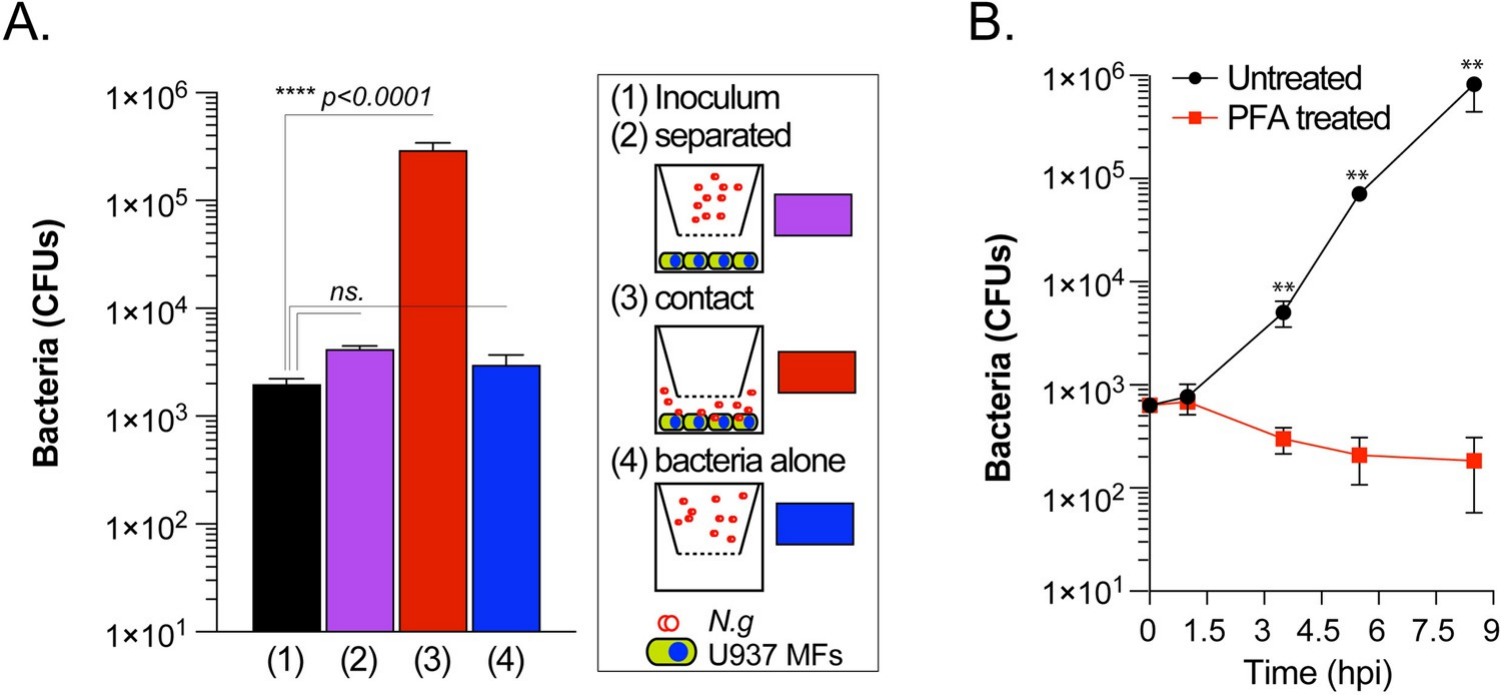
Direct contact with live macrophages is required for gonococcal replication. **(A)** *N*.*g* FA1090 growth under conditions that permit or restrict direct contact with U937 MFs in PBSG media. Contact separation between bacteria and the macrophages is facilitated by a 0.4μm filter barrier. Total CFUs recovered at 8hpi are shown. **(B)** *N*.*g* FA1090 growth in association with PFA-fixed U937 MFs in PBSG media. Macrophages were fixed for 45min with PFA (4% vol./vol. in PBS) and extensively washed with warm PBS prior to infection. Total CFUs recovered at the indicated time-points are shown. **(A-B)** Representative experiments from two (B) or three (A) biological replicates are included. **(A-B)** For each condition means of technical triplicates ± SD are shown, **(A)** one-way ANOVA analysis, not significant (ns.); **(B)** two-way ANOVA, ** p = 0.0016.

The rate of gonococcal replication in association with human macrophages in PBSG (nutrients-poor media) exceeded the growth rate observed in the nutrients-rich complex gonococcal base liquid (GCBL) medium (Fig 1A and 1B), indicating that somehow gonococci harvest nutrients from macrophages. When gonococci were separated from the U937 MFs with a 0.4μm pore size filter barrier that allowed fluid exchange but prevented direct contact, gonococci did not replicate (Fig 2A). Similarly, macrophages fixed with paraformaldehyde prior to infection did not support gonococcal replication (Fig 2B), indicating that direct contact with live macrophages is required for acquisition of the nutrients needed for gonococcal replication. Together, these data demonstrate that gonococci encode the capacity to colonize and replicate in association with human macrophages.

### *N.g* occupies multiple distinct cellular niches on human macrophages

**Fig 3:**
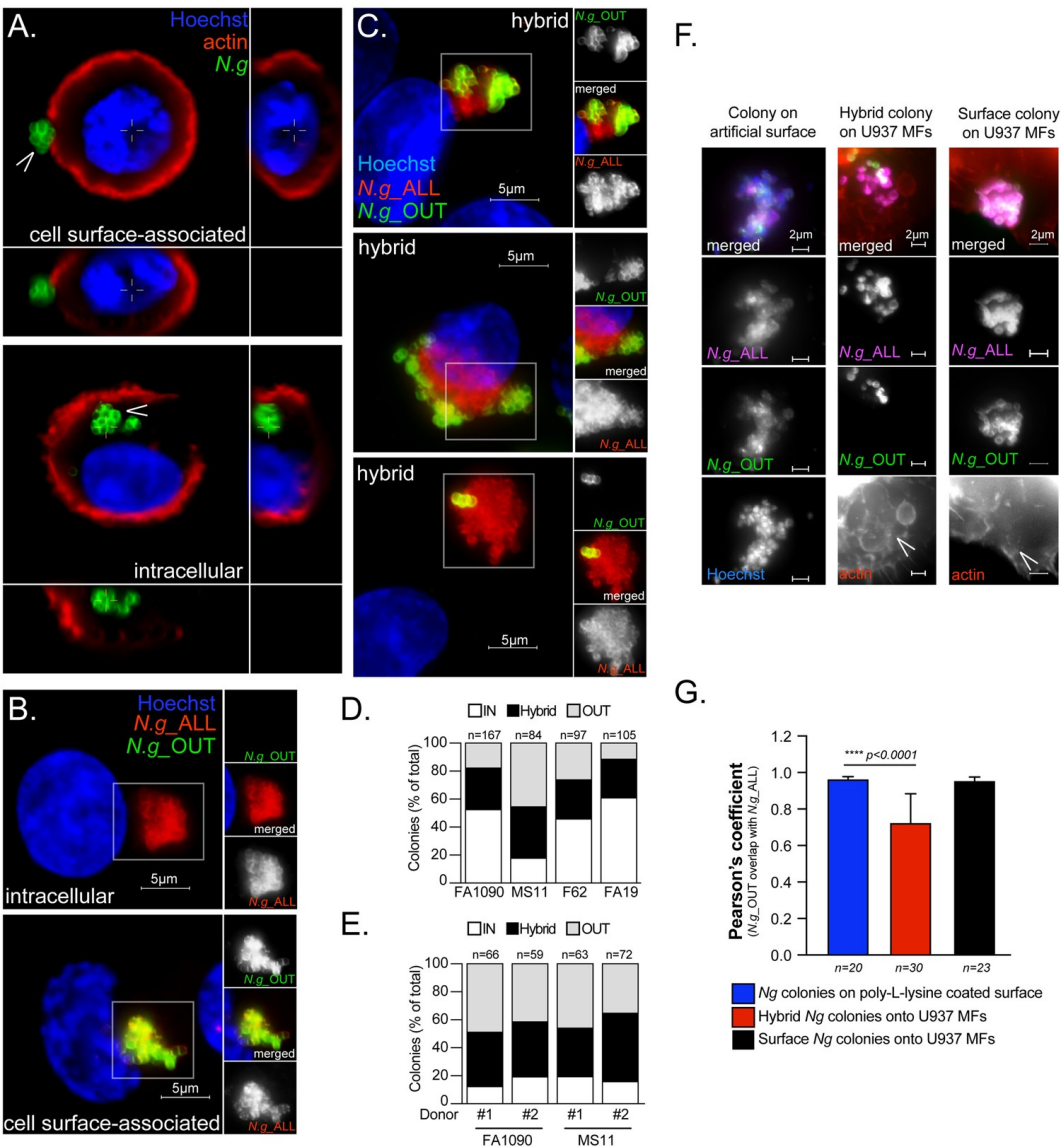
Gonococci colonize distinct cellular compartments. **(A-C)** U937 MFs infections with *N*.*g* FA1090 at MOI = 2. **(A)** Representative single focal plane of 3D image Z-stacks showing intracellular and cell-surface associated gonococcal colonies at 6 hpi. Side panels show Z-axis reconstruction at the indicated cross-sections. **(B-C)** Micrographs of colonized macrophages after inside/out staining with anti-*N*.*g* antibodies. **(B)** Single-stained intracellular (red) and double-stained surface-exposed (yellow) gonococcal colonies are shown. **(C)** Multiple representative partially exposed *(hybrid)* gonococcal colonies (yellow and red) are shown. **(D-E)** Quantitative analyses of the relative colony topologies distributions from infections with different *N.g* strains at 10hpi **(D)** in U937 MFs and **(E)** in human primary monocyte-derived macrophages (hMDMs) derived from two distinct donors are shown. **(F)** Representative z-projections micrographs of *N*.*g* colonies formed on artificial surface (poly-L-lysine coated glass coverslips) or in association with U937 MFs that were stained using the inside/out methodology. **(G)** Analysis of *N*.*g*_OUT and *N*.*g*_ALL signal colocalization using Pearson’s correlation coefficient (PCC) in each of the three *N*.*g* colony types represented in **(F)**. Means ± SD are shown, unpaired T-test analysis. **(A-G)** Representative experiments from two **(E-G)** or at least three **(A-D)** biological replicates are included. **(D-E, G)** The number of colonies analyzed from each condition is indicated by “*n*”.

The cellular niche in macrophage infections that supports gonococcal replication remains unknown. In previous studies [12], intracellular as well as cell surface-associated gonococci have been observed. Thus, we investigated the subcellular localization of gonococcal colonies because those cellular compartments likely include the niche that supports bacterial replication. In this study, groups of 4–12 bacteria are referred to as a *microcolony* and groups larger than 13 members are referred to as a *colony*. A 3D immunofluorescence microscopy analysis of U937 MFs infected with *N*.*g* FA1090 for 6 hours was performed, where the cortical actin network was labeled with phalloidin (Fig 3A). Cortical actin can discern intracellular from cell-surface localized bacteria as it is formed in close proximity to the plasma membrane. Based on this criterion, both intracellular and cell surface-associated gonococcal colonies were observed (Figs 3A and S2A–S2D). These distinct colony topologies were confirmed by an alternative inside/out immunofluorescence microscopy approach, in which extracellular bacteria are labeled with two distinct polyclonal anti-*N*.*g* antibodies (one used prior to and another used after cell permeabilization), whereas intracellular bacteria are single-labeled because they are accessible only after cell permeabilization (Fig 3B). Interestingly, mosaic colonies consisting of single antibody-stained as well as dual-stained regions within the same colony were frequently observed (Fig 3C). This hybrid colony type was produced by all four gonococcal strains tested (FA1090, MS11, F62, and FA19) in U937 MFs infections (Fig 3D) as well as in infections of human primary monocyte-derived macrophages (hMDMs) (Figs 3E and S2C and S2D). These data demonstrate that gonococcal colonies were not exclusively intra- or extracellular but rather occupied multiple distinct cellular niches on infected human macrophages by a mechanism conserved in primary hMDMs and U937 MFs.

**Fig 4:**
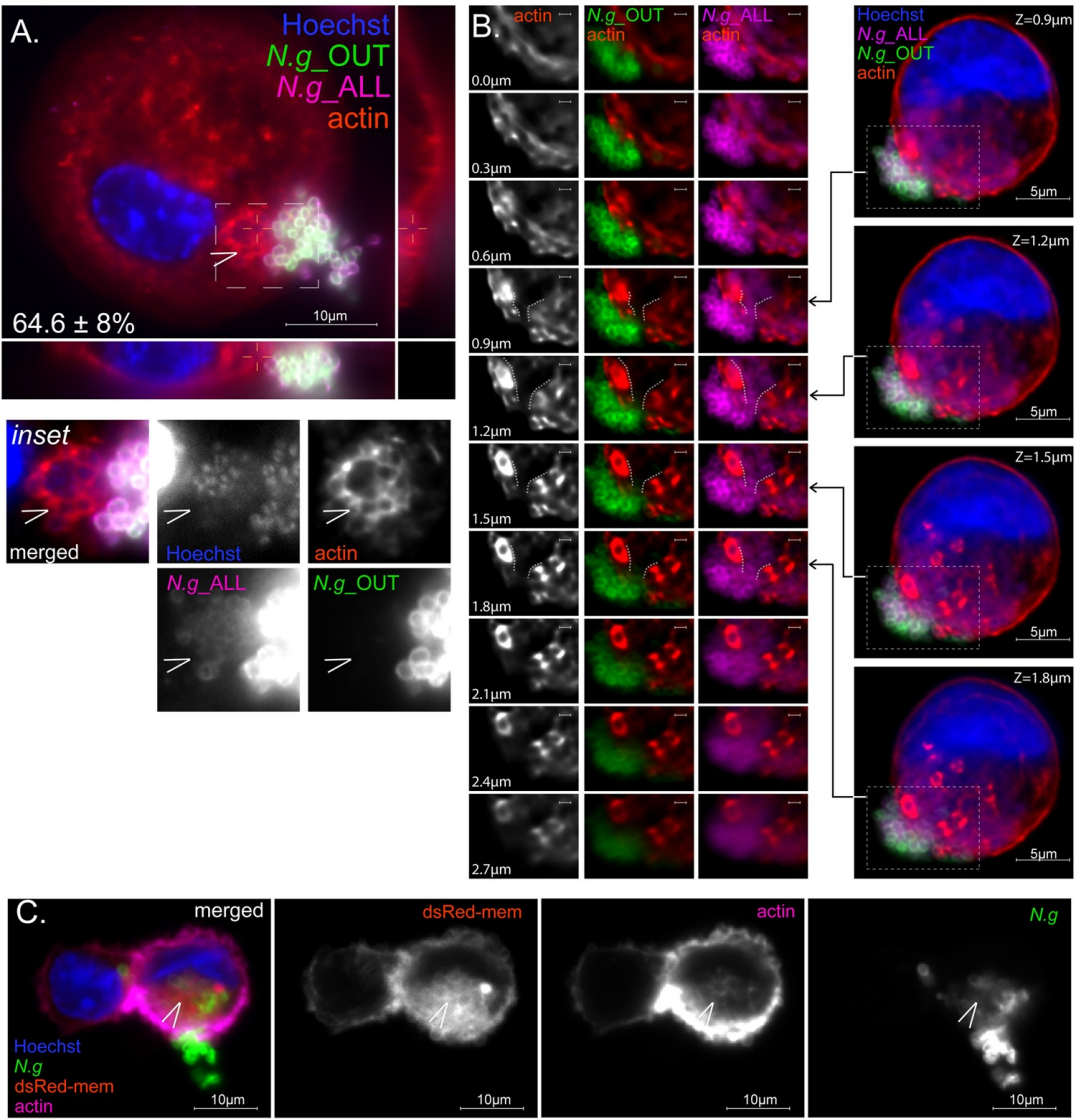
Actin microfilaments and membrane invaginations localize at plasma membrane sites colonized by hybrid gonococcal colonies. Micrographs show representative embedded gonococcal colonies on U937 MFs (MOI = 2) at 8hpi **(A)** and 10hpi **(B)** after inside/out differential staining. **(A)** Side panels show Z-axis reconstruction of the indicated cross-section. Inset shows polymerized actin accumulation (observed on 64.6 ± 8% of embedded colonies, n = 54) specifically on the intracellular region of the hybrid colony (arrowhead). **(B)** Embedded colonies are continuous. A series of single focal planes spanning 2.7μm shows that the transition point—from intracellular to extracellular region of an embedded colony is continuous and bracketed by cortical actin highlighted by the dashed lines. **(C)** Actin-rich plasma membrane invaginations surround the intracellular region of hybrid colonies (arrowhead). Single focal plane micrograph of a U937 MF stably expressing the plasma membrane-associated fluorescent reporter DsRed-mem and harboring a hybrid gonococcal colony. **(A-C)** Representative experiments from at least three biological replicates are included.

We considered that the hybrid colony topology could represent a surface-associated colony in which tightly packed outer layer gonococci render innermost bacteria inaccessible to antibody binding. However, this was not the case because inside/out immunostaining of *N*.*g* colonies on poly-L-lysin coverslips in the absence of macrophages show that majority of bacteria are accessed equally well by both antibodies (Fig 3F and 3G). We noticed that in colonies with hybrid topology the antibody inaccessible regions penetrate the cortical actin layer and are enveloped by a meshwork of actin filaments (Fig 4A). Moreover, hybrid colonies remain continuous and appear as a single entity even though a clear demarcation between the antibody accessible and inaccessible colony regions is evident by inside/out microscopy (Fig 4B). The most plausible explanation for the hybrid topology is partial internalization, where the extracellular gonococci acts as a plug to seal a plasma membrane invagination harboring the intracellular portion of the colony. We transduced U937 MFs with the genetically encoded fluorescent membrane marker dsRed-mem. Posttranslational palmitoylation of Cys3 and Cys4 targeted dsRed-mem predominantly to the plasma membrane of U937 MFs. Indeed, plasma membrane invaginations co-localize with the actin meshwork at the cell-proximal regions of hybrid gonococcal colonies (Fig 4C). Together, these data indicate that on colonized human macrophages gonococcal colonies can be found: (i) on the cell surface, (ii) within an intracellular compartment and (iii) partially internalized within in a plasma membrane invagination.

### Intracellular gonococcal colonies arise from prolonged internalization of surface-associated gonococcal microcolonies

**Fig 5:**
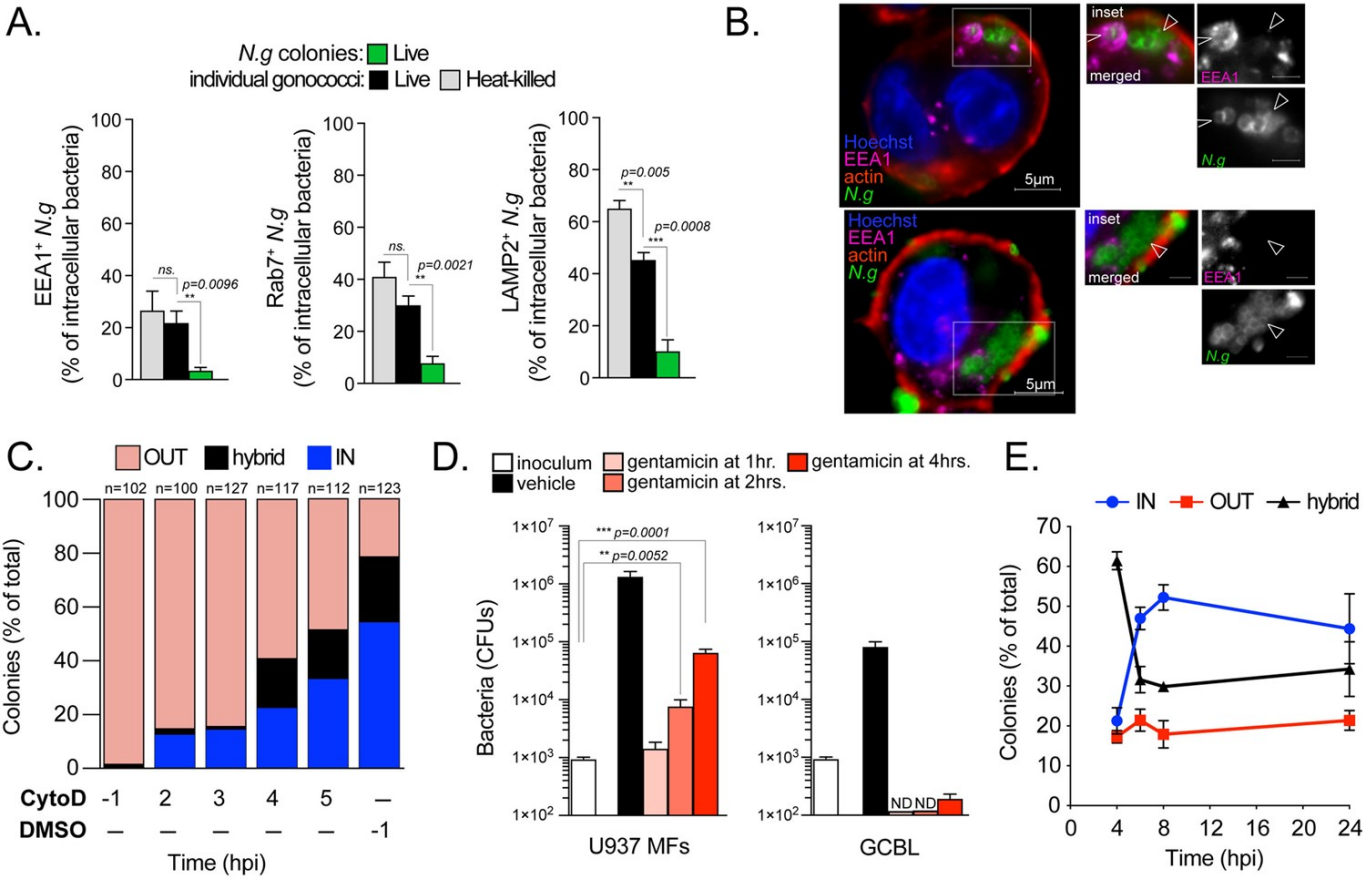
Trafficking and kinetics of intracellular colonization by gonococci. **(A-B)** Analysis of endosomal maturation of *N*.*g* FA1090-occupied phagosomes. **(A)** Recruitment of early (EEA1) and late endosomal (Rab7 and LAMP2) markers to phagosomes containing heat-killed or live individual gonococci at 2hpi and internalized gonococcal colonies at 6hpi. At least 100 bacteria or bacterial colonies were analyzed for each condition. **(B)** Representative single focal plane micrographs showing EEA1 accumulation on intracellular diplococci (arrowhead) and EEA1 exclusion from internalized gonococcal colonies (triangles). **(C)** Late entry facilitates intracellular colonization. Colony types distribution analysis of U937 MFs infected with *N*.*g* FA1090 at 8hpi when the actin polymerization inhibitor cytochalasin D (5 μM) was added at the indicated time points to block bacterial internalization. Bacterial internalization is blocked even when cytochalasin D is added as late as 3hpi. **(D)** *N*.*g* survives intracellularly in U937 MFs upon late gentamicin treatment. Bacterial CFUs recovered at 8hrs following a gentamicin protection assay in infected U937 MFs or in liquid culture (GCBL) are shown. Gentamicin (4μg/ml) was added at the indicated time-points. **(E)** Kinetic analysis of colony types in U937 MFs infections (MOI = 2) with *N*.*g* FA1090. At least 80 colonies for each condition were scored. **(A, D and E)** Means of technical triplicates ± SD are shown, unpaired T-test, not significant (ns.). **(A-E)** Representative experiments from two **(C)** or at least three **(A-B, D-E)** biological replicates are included.

In the early time points (< 2hpi), macrophage associated gonococci were predominantly diplococcus (>90%) (S3A Fig). Large percentage of those bacteria were internalized (S3B Fig) and resided within the endolysosomal compartment as indicated by the recruitment of the early (EEA1) and late endosomal (Rab7 and Lamp2) markers (Figs 5A and 5B and S6B and S6C). Similar internalization and trafficking kinetics were seen in macrophage infections with heat-killed gonococci (Figs 5A and S3B). Because lysosomes are degradative organelles that promote clearance of intracellular gonococci in U937 MFs infections [12], it is unlikely that early entry events give rise to the intracellular colonies observed at later time points. To determine when successful intracellular colonization is initiated, we blocked phagocytosis by adding the actin polymerization inhibitor cytochalasin D at discrete times post infection and scored colony localizations at 8 hpi (Fig 5C). Pre-treatment with cytochalasin D, as expected, mostly resulted in surface-associated colonies. However, blocking phagocytosis at 2hpi markedly reduced the number of intracellular colonies detected at 8hpi by 74% (from 53% to 14%). Even as late as 4hpi cytochalasin D addition reduced intracellular colonies by >60% (Fig 5C). These results further support the notion that rapidly phagocytosed diplococci (< 2hpi), which are transported to lysosomes (Fig 5A), do not give rise to intracellular colonies and demonstrate that productive intracellular colonization is largely carried out by bacteria internalized at later times (> 2hpi). An alternative approach using gentamicin (an aminoglycoside antibiotic that cannot diffuse across biological membranes) protection assays produced similar results of complete sensitivity to the drug at 1hpi and a significant increase in the protected bacterial population from 2hpi onwards (Fig 5D). The number of intracellular bacteria might be underestimated given that gentamicin can be internalized by pinocytosis and likely kill a fraction of intracellular gonococci [67]. Together, these data argue that intracellular colonization is primarily carried out by gonococci that initially remain surface associated for two or more hours prior to entry.

To define when and where gonococci initiate replication, we investigated the trafficking kinetics of gonococcal microcolonies (4–12 bacteria) as a readout for initiation of bacterial replication. Microcolonies were detectable at 2 hpi when macrophages were infected with live but not heat-killed gonococci (S3A Fig); however, majority of cell-associated bacteria were diplococci (~ 90%) and only a few macrophages harbored bacteria at that time (Fig 1C). At 2hpi, the majority of microcolonies were at least partially surface-associated (> 87%) (S3C and S3D Fig). From 2 to 4hpi, the percentage of intracellular microcolonies increased (from 13% to 33%) (S3C Fig). Taken together these data suggest that intracellular colonies are seeded by bacteria that undergo one or more rounds of replication at the macrophage surface to form a colony prior to internalization. In agreement, the percentage of hybrid and intracellular colonies inversely correlated over time, where colonies with hybrid topology were the most prevalent colony type at 4hpi but decreased by 50% at 8hpi concomitant with a sharp increase in intracellular colonies (Fig 5E). Importantly, this relationship depended upon unperturbed internalization (Fig 5C).

A fraction of gonococcal colonies (~20%) were strictly surface-associated (like in Fig 3B) when quantified at discrete time points in a 24hrs infection (Fig 5E). Because of the high phagocytic rate, persistence of large strictly surface-associated colonies is surprising given that colonized U937 MFs internalized efficiently both opsonized IgG-coated beads as well as non-opsonized heterologous bacterial cargo (S4 Fig). The most plausible explanation for these data is that some gonococcal colonies resist phagocytosis, remain surface-associated and expand. Indeed, prolonged phagocytosis blockade with cytochalasin D did not reduce the rate of bacterial replication and resulted in large surface-associated gonococcal colonies on macrophages (S5A and S5B Fig), demonstrating that gonococci can replicate on the macrophage plasma membrane independent of internalization.

Next, we investigated whether in macrophage infections gonococci replicate intracellularly because large intracellular colonies could be produced in principle strictly through internalization of large surface colonies. To this end, we measured the change in size of intracellular colonies from 4 to 10hpi under conditions that either blocked or did not block bacteria uptake at 4hpi (S5C and S5D Fig). The mean volume of intracellular colonies increased ~ 4-fold from 4 to 10hpi irrespective of cytochalasin D treatment (S5D Fig); even though cytochalasin D blocked continuous bacteria uptake as indicated by the significant reduction in intracellular colonies (S5C Fig). Thus, intracellular gonococcal colonies continue growing even after internalization demonstrating that in macrophage infections gonococci can establish two distinct cellular niches which support bacterial replication—one is intracellular and another is cell surface-associated.

### A unique actin-enriched membrane-bound organelle harbors replicating intracellular gonococci in macrophage infections

**Fig 6:**
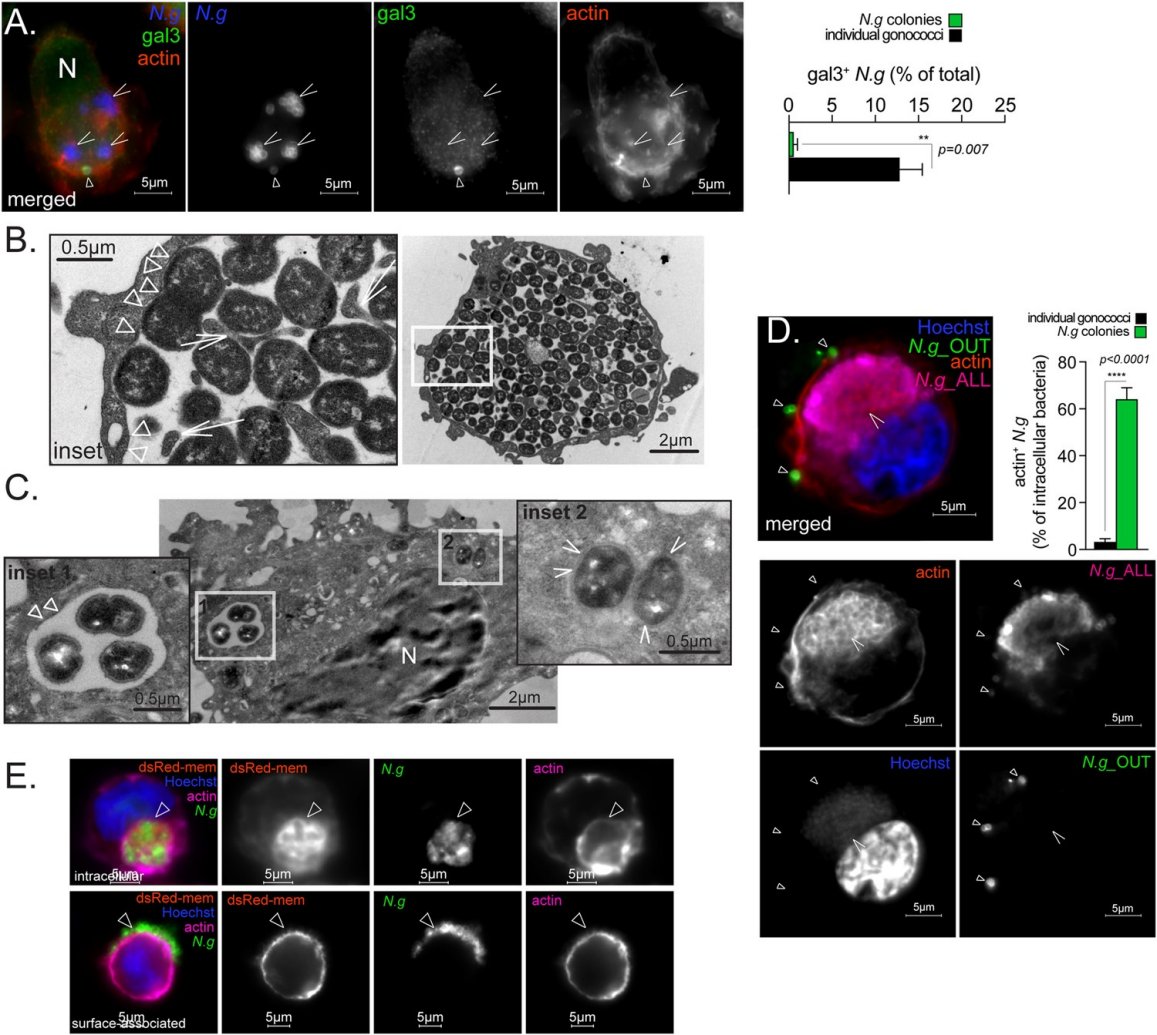
Gonococcal colonies evade endocytic maturation upon entry in macrophages and occupy a unique membrane-bound organelle surrounded by actin filaments. **(A)** Representative single focal plane micrograph showing galectin-3 recruitment to some intracellular gonococcus (triangle) but not to gonococcal colonies (arrowhead). Quantitative analysis of galectin-3 recruitment to intracellular gonococci at 4hpi. **(B-C)** Representative TEM micrographs showing large **(B)** and small **(C)** intracellular gonococcal colonies that are separated from the cytosol by a membrane (insets, triangles). Some diplococci localize in the cytosol (inset, arrowheads). **(B)** Intralumenal membrane invaginations in close contact with gonococci within the *N*.*g*-occupied vacuole are denoted with arrows in the inset. **(D)** Polymerized actin filaments surround intracellular gonococcal colonies in U937 MFs infected by *N*.*g* FA1090. Quantitative analysis and representative single focal plane micrographs showing actin accumulation on an intracellular colony (arrowhead) at 10hpi. Triangles point to surface-associated diplococci. Individual channels of the merged image are shown in grayscale. **(E)** Representative single focal plane micrographs of U937 MFs harboring internalized or surface-associated colonies. Intracellular gonococcal colonies retain the plasma membrane marker DsRed-mem. **(A and D)** Means of technical triplicates ± SD are shown, unpaired T-test. At least 100 individual gonococci or gonococcal colonies were analyzed for each condition. **(A-E)** Representative experiments from two **(B-C)** or at least three **(A, D-E)** biological replicates are included.

The intracellular niche supporting gonococci replication in macrophage infections remains unknown, although it has been speculated that it takes place in cytosol [12]. Thus, we first sought experimental evidence for cytosolic replication by measuring the recruitment of the cytosolic β-galactoside-binding lectin galectin-3 to intracellular gonococcal colonies. Galectin-3 is frequently used for identification of cytosol invading bacteria because it binds sugar moieties present on the bacterial surface as well as on luminal glycoproteins that can be accessed by galectin-3 upon vacuolar rupture [68,69]. Only a minor fraction of intracellular diplococci accumulated galectin-3 in U937 MFs whereas intracellular gonococcal colonies did not (Fig 6A), indicating that colonies are somehow protected from cytosolic lectins even though galectin-3 clearly can bind *N*.*g*. To determine whether a membrane separates gonococcal colonies from the cytosol, we performed transmission electron microscopy of colonized macrophages, which confirmed that colonies of different sizes occupied membrane-bound vacuolar compartments (Fig 6B and 6C). Intralumenal membrane invaginations in contact with gonococci were also observed within the large gonococci-containing vacuoles (Fig 6B). Consistent with the galectin-3 recruitment data, some diplococci were found in direct contact with the cytosol (Fig 6C). To investigate intravacuolar localization of gonococcal colonies we used U937 MFs transduced with fluorescent membrane marker dsRed-mem. In colonized macrophages, intracellular colonies were enveloped by dsRed-mem confirming that a membrane-bound organelle harbored the bacteria (Fig 6E).

To gain insight into the biogenesis of the gonococci-occupied organelle, we investigated the endosomal maturation of phagosomes containing gonococcal colonies. Phagosomes containing either diplococci or heat-killed gonococci rapidly acquired endosomal identity and accumulated early (EEA1) as well as late (Rab7 and LAMP2) endosomal markers unlike internalized gonococcal colonies (Figs 5A and 5B and S6A–S6E). Instead, an extensive actin filaments network surrounded intracellular gonococcal colonies within U937 MFs (Fig 6D) and primary hMDMs (S2C Fig and S1 Video). The actin microfilaments network begun forming during colony internalization (Fig 4A) and is retained around majority of intracellular colonies as well (Fig 6D). The persistence of actin polymerization around internalized gonococcal colonies is inconsistent with the actin dynamics during phagocytosis as actin networks surrounding nascent endosomes are rapidly disassembled [70]. Retention of dsRed-mem onto internalized colonies (Fig 6E) combined with inability to recruit endosomal markers (Fig 5A and 5B) indicates that the gonococci occupied vacuole avoids fusion with the endocytic compartment and somewhat retains plasma membrane characteristics.

### Gonococcal colonies invade human macrophages via a mechanism mediated by formin-dependent actin polymerization

**Fig 7:**
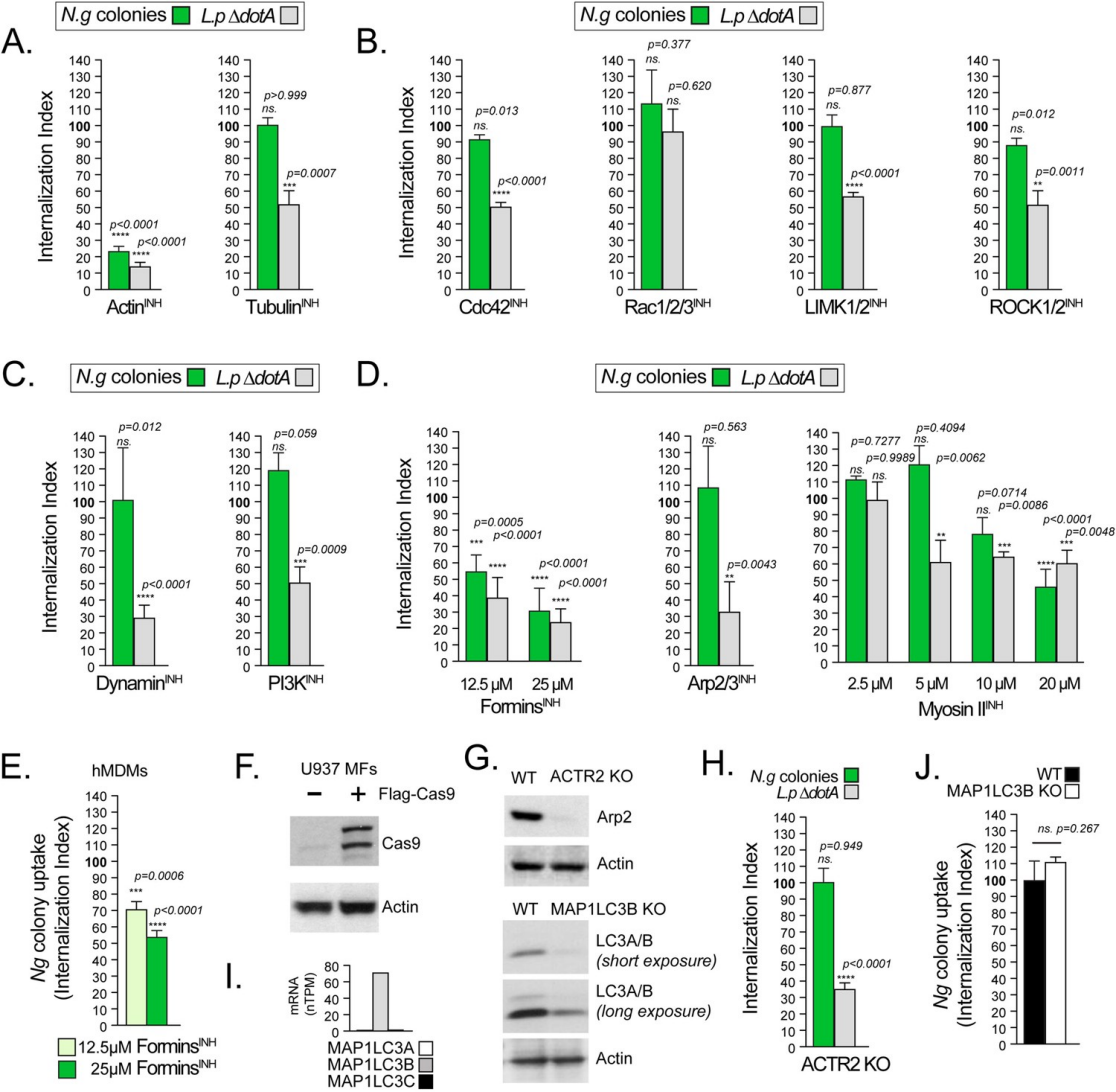
Gonococci enter macrophages despite a block in phagocytosis. **(A-E)** Gonococci internalization by U937 MFs **(A-D, H and J)** and hMDMs **(E)** requires formin-mediated actin polymerization but not canonical phagocytosis. **(A-E, H and J)** Quantitative analysis of bacteria uptake by macrophages using inside/out microscopy in the presence/absence of different inhibitors added 30 min prior to infection. Gonococcal infections were stopped at 8hpi and the percentage of intracellular colonies in each condition was calculated (green bars). *L*. *pneumophila ΔdotA* infections were stopped at 2hpi and the percentage of internalized bacteria was determined (gray bars). The **internalization index** reflects how each treatment affects uptake compared to vehicle control and was calculated for each condition by dividing the percentage of internalized objects from the treatment condition by the percentage of internalized objects from the respective vehicle control condition, which was then multiplied by 100. Inhibitors targeting: **(A)** actin polymerization (cytochalasin D, 5μM) and microtubules polymerization (nocodazole, 3μM); **(B)** the small GTPases Cdc42 (ML-141, 5μM), Rac1/2/3 (EHT 1864, 5μM) and the downstream effectors of Rho GTPase—LIMK1/2 kinases (BMS-5, 1μM) and ROCK1/2 kinases (GSK269962, 1μM); **(C)** the phagosome regulators Dynamin (dynasore, 30μM) and Phosphoinositide-3 kinase (LY294002, 10μM); **(D)** actin polymerization regulators formins (SMIFH2, 12.5 or 25μM), Arp2/3 complex (CK869, 10μM), Myosin II ATPases ((-) blebbistatin, from 2.5 to 20μM). **(E)** Decreased gonococci uptake at 8hpi by hMDMs treated with SMIFH2 as indicated. **(F)** Immunoblot analysis of the U937 cell line stably expressing Cas9 used for CRISPR genome editing. **(G)** Immunoblot validation for the loss of Arp2 in the *ACTR2* KO and LC3 in the *MAP1LC3B* KO U937 MFs. **(H and J)** *N*.*g* colony internalization is not affected by the loss of **(H)** Arp2 or **(J)** LC3. **(I)** mRNA expression for the indicated genes in U937 cells from RNAseq presented in normalized transcript per million (nTPMs). Data obtained from the Human Atlas Project [112]: MAP1LC3A (https://www.proteinatlas.org/ENSG00000101460-MAP1LC3A/cell+line); MAP1LC3B (https://www.proteinatlas.org/ENSG00000140941-MAP1LC3B/cell+line); MAP1LC3C (https://www.proteinatlas.org/ENSG00000197769-MAP1LC3C/cell+line). **(A-E, H and J)** Means from at least three biological replicates ± SD. At least 100 *N*.*g* colonies or *L*.*p ΔdotA* bacteria for each condition were scored. The statistical significance of the differences between the internalization index from inhibitor-treated cells and the vehicle-treated cells for each condition were calculated using the unpaired T-test **(A-C, D for Arp2/3INH, H and J)** or one-way ANOVA **(D** for **Formin**^**INH**^ and **MyosinII**^**INH**^**, E)**, not significant (ns.).

The prolonged entry kinetics as well as the absence of endosomal markers on the *N*.*g* colony-containing vacuoles are inconsistent with canonical phagocytosis-dependent uptake and endosomal transport [71–73] and prompted us to investigate the colony internalization mechanism. To this end, different host factors were targeted pharmacologically to identify molecular determinants of *N*.*g* colony uptake by macrophages. Colony internalization was measured using inside/out microscopy in U937 MFs at 8hpi (Fig 7). In parallel controls we measured the phagocytic uptake of a heterologous live bacterial cargo by culturing U937 MFs with non-pathogenic *Legionella pneumophila ΔdotA* (*L*.*p* Δ*dotA*) bacteria, which are internalized via non-specific phagocytosis and are transported to lysosomes through the endosomal pathway within minutes [74]. As expected, internalization of both *L*.*p* Δ*dotA* and *N*.*g* colonies required actin polymerization (Fig 7A, Actin^INH^). Depolymerization of microtubules with nocodazole did not affect *N*.*g* colony uptake but partially reduced phagocytosis of *L*.*p* Δ*dotA* (Fig 7A, Tubulin^INH^). Similarly, *N*.*g* colonies were internalized efficiently when the Rho family of small GTPases were inhibited, whereas *L*.*p* Δ*dotA* phagocytosis was significantly reduced (Fig 7B). Phagocytosis of *L*.*p* Δ*dotA* by U937 MFs required functional Cdc42 as well as the RhoA GTPase effector kinases ROCK1/2 and LIMK1/2, which trigger actin polymerization to shape the phagocytic cup and mediate cargo internalization [75], whereas Rac1/2/3 functions were dispensable (Fig 7B). Phagosome closure and scission processes regulated by PI3K [76] and dynamin [77] were also required for *L*.*p* Δ*dotA* uptake (Fig 7C). However, gonococcal colonies were internalized efficiently by U937 MFs even when Rho GTPases, PI3K and dynamin were inhibited (Fig 7B and 7C), demonstrating that *N*.*g* colonies are internalized by macrophages via an actin-dependent mechanism distinct from canonical phagocytosis.

Three main actin nucleator machineries regulate membrane morphogenesis–(i) the formin family members extend linear nuclear filaments [78]; (ii) the Arp2/3 complex nucleates branched actin filaments [79]; (iii) Myosin II ATPases regulate the contractile actomyosin bundles [65]. The phagocytic uptake of *L*.*p* Δ*dotA* depended upon all three, as expected (Fig 7D and 7E). Interestingly, gonococcal colony internalization was not affected by Arp2/3^INH^ (Fig 7D). To confirm that the Arp2/3 complex is dispensable for *N*.*g* colony internalization using an alternative approach, we utilized CRISPR/Cas9 genomic editing to generate U937 MFs in which the *ACTR2* gene encoding Arp2 is inactivated (Fig 7F and 7G). Despite a general defect in phagocytosis caused by loss of Arp2, *ACTR2* KO U937 MFs internalized gonococcal colonies as efficiently as the parental cell line (Fig 7H). In contrast, the formins inhibitor SMIFH2 [78] treatment significantly decreased colony uptake in a dose dependent manner in U937 MFs (Fig 7D, Formins^INH^) and in hMDM (Fig 7E) infections. Importantly, the SMIFH2 treatment doses we used do not decrease U937 MFs viability (S7 Fig). The Myosin II^INH^ at 5μM achieved maximal reduction in *L*.*p* Δ*dotA* uptake, whereas *N.g* colony internalization was not affected (Fig 7D). However, increasing the Myosin II^INH^ dose to 20 μM resulted in significant defect in *N*.*g* colony uptake indicating that actomyosin contractility regulates the actin cytoskeleton during *N*.*g* colony internalization (Fig 7D).

LC3-associated phagocytosis (LAP) is a cellular trafficking pathway that incorporates the cell autophagic machinery to conjugate LC3 family proteins to the phagosomal membrane, thus facilitating phagosome maturation and transport of various cargos (including pathogens) to lysosome for degradation [80]. To determine whether LAP or canonical autophagy regulates *N*.*g* internalization we generated MAP1LC3B KO U937 MFs (Fig 7G). U937 cells express MAP1LC3B, which encodes LC3B, but not MAP1LC3A and MAP1LC3C (Fig 7I). Immunoblot analysis with α-LC3A/B antibody confirmed that LC3A is undetectable when LC3B is knockout (Fig 7G) confirming that LC3B is the principle LC3 in U937 MFs. Inactivation of LC3B did not affect *N*.*g* colony internalization (Fig 7J) indicating that LAP and autophagy are unlikely to be significant regulators of this process. In agreement, LC3 was only recruited to cytosolic gonococci (S6F Fig) and not to invading *N*.*g* colonies.

Thus, gonococci directly or indirectly trigger a plasma membrane morphogenesis mechanism distinct from canonical phagocytosis that facilitates macrophage invasion via the production of linear actin filaments mediated by one or more members of the formins family. The dynamic behavior of these actin filaments that results in *N*.*g* colony internalization is likely mediated in part by the forces generated by myosin motor proteins.

### FMNL3 localizes at sites of gonococcal invasion and mediates *N.g* colony internalization in human macrophages

**Fig 8:**
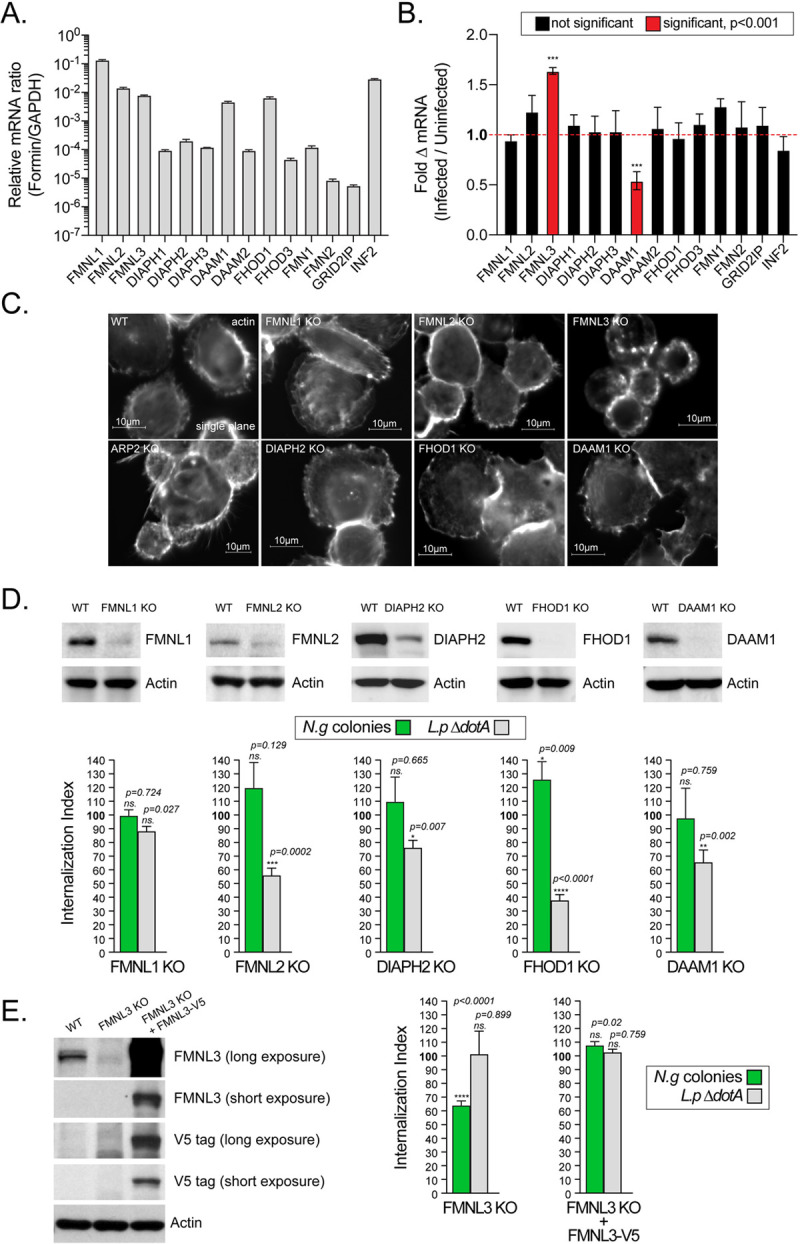
FMNL3 mediates invasion of gonococcal colonies in macrophages. **(A-B)** qPCR mRNA expression analyses for the members of the formin family in U937 MFs are shown. mRNA abundance relative to *gapdh* mRNA is shown in **(A)**. Fold change in mRNAs abundance upon *N*.*g* FA1090 infection of U937 MFs at 4 hpi is shown in **(B). (A-B)** Means of three biological replicates ± SD are shown, *** p < 0.001 unpaired T-test **(C)** Representative single plane micrographs of differentiated resting U937 MFs cell lines stained with phalloidin. **(D and E)** Analysis of gonococcal colony internalization, phagocytosis and immunoblot validation of U937 MFs cell lines lacking different formins. **(D and E)** The Internalization index for each condition was calculated by dividing the percentage of internalized objects from infections of the knock out U937 MFs cell line by the percentage of internalized objects from infections of the parental U937 MFs, which was then multiplied by 100. Values > 100% indicate increased object internalization by the KO cell line compared to WT cells, whereas values < 100% indicate decreased object internalization by the KO cell line. At least 100 objects for each condition were scored. Means from three biological replicates ± SD. The statistical significance of the differences between the internalization index from the KO cells and the parental cells for each condition were calculated using the unpaired T-test, not significant (ns.). **(D and E)** Immunoblot validation for loss of protein expression in U937 MFs differentiated from several KO cell lines in which different formin genes were inactivated. Complementation of the FMNL3 KO U937 MFs with V5-tagged FMNL3 is shown in **(E)**.

The formin family of actin nucleators consist of fifteen members [81]–all family members except FHDC1 were expressed by U937 MFs to various degrees (Fig 8A). The mRNAs for FMNL1, FMNL2, FMNL3, DAAM1, FHOD1 and INF2 were the most abundantly expressed. Gonococcal infection did not alter the mRNA abundance of the formins family members with the exception of FMNL3 and DAAM1 (Fig 8B). At 4 hpi the FMNL3 mRNA increased by ~50% whereas DAAM1 mRNA abundance decreased by ~50% (Fig 8B). To determine which formin mediates gonococcal invasion we utilized a genetic approach to knock out individual members in U937 cells using CRISPR/Cas9 genome editing. The following U937 cell lines were successfully generated and validated via immunoblot analysis: FMNL1 KO, FMNL2 KO, FMNL3 KO, DAAM1 KO, FHOD1 KO, DIAPH2 KO (Fig 8C–8E). Next, the capacity of the individual U937 MFs knock out cell lines to phagocytose *L*.*p ΔdotA* and to internalize gonococcal colonies was compared to the parental cell line. Deletion of FMNL1 did not affect significantly uptake of either *L*.*p ΔdotA* or gonococcal colonies; however, loss of FMNL2, DAAM1 and DIAPH2 significantly reduced *L*.*p ΔdotA* phagocytosis by 45%, 35% and 20% respectively yet gonococcal colony were internalized efficiently (Fig 8D). In the absence of FHOD1, macrophage phagocytosis of *L*.*p ΔdotA* was reduced by 65% but the number of internalized gonococcal colonies increased significantly by 20% (Fig 8D). Conversely, loss of FMNL3 significantly reduced gonococcal invasion by 40% despite normal *L*.*p ΔdotA* phagocytosis (Fig 8E). Expression of C-terminally V5 tagged FMNL3 allele in the *FMNL3* KO U937 MFs fully complemented the defect in gonococcal colony internalization (Fig 8E). It’s notable that FMNL3-V5 overexpression did not increase colony uptake compared to WT U937 MFs which express much lower amounts of FMNL3 (Fig 8E) indicating that endogenous levels of FMNL3 are sufficient for maximal uptake and the 50% increase in the FMNL3 transcripts induced by gonococcal infection (Fig 8B) might not be biologically meaningful. Together, these results demonstrate that macrophage invasion by gonococci is mediated by FMNL3, a formin that does not regulate phagocytosis in U937 MFs, whereas the formins that impact macrophage phagocytosis (FHOD1, FMNL2, DAAM1 and DIAPH2) were dispensable for colony internalization.

**Fig 9:**
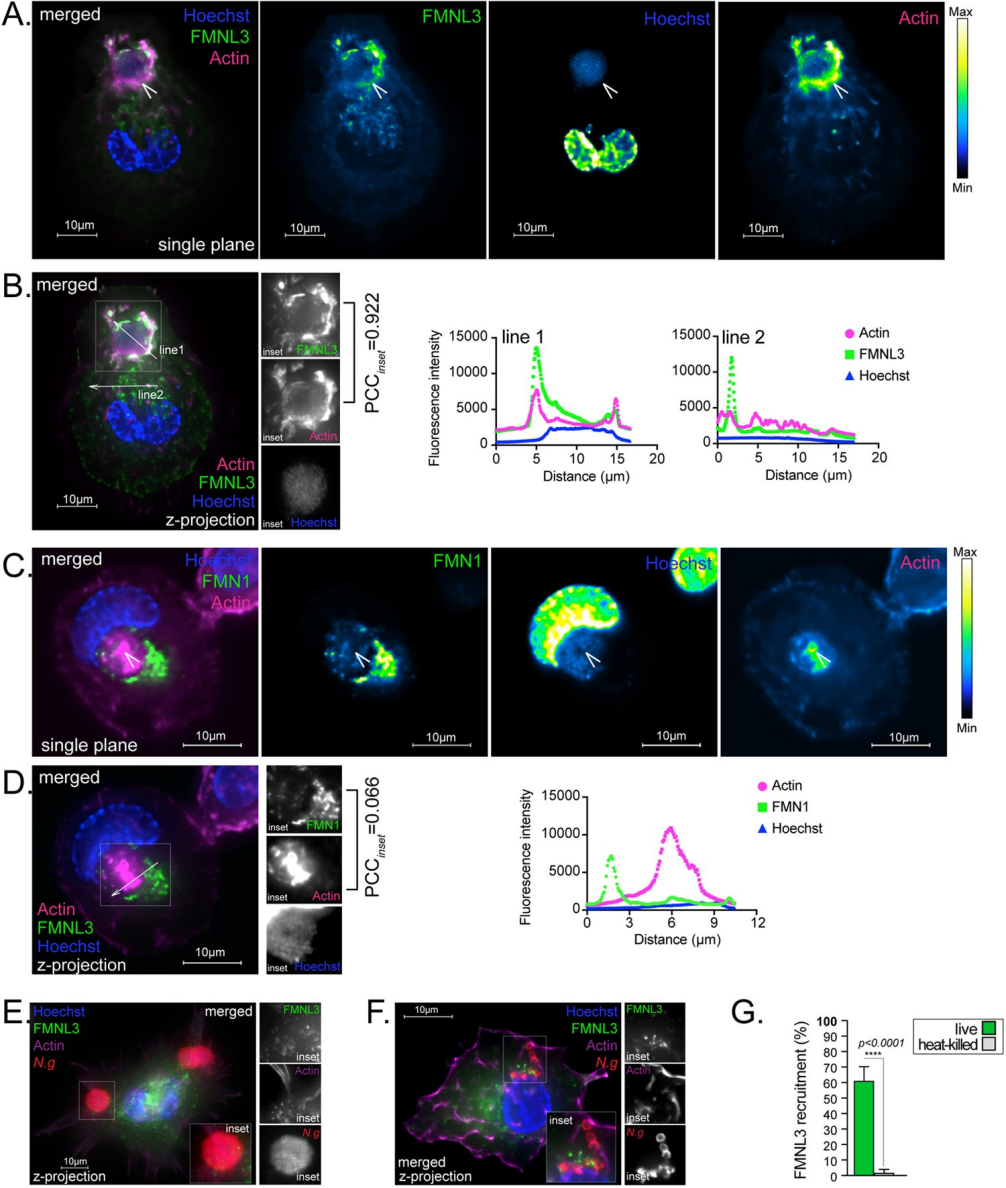
FMNL3 co-localizes with polymerized actin at the sites of N.g colony plasma membrane invasion. Micrographs show U937 MFs expressing FMNL3-V5 **(A-B, E-F)** or FMN1-V5 **(C-D). (A-D)** Representative micrographs showing FMNL3-V5 **(A-B)** but not FMN1-V5 **(C-D)** localizes at the actin ruffles at the plasma membrane surrounding an invading *N*.*g* FA1090 colony. Single plane image is shown in **(A and C)** from the z-projection image shown in **(B and D)**. Individual channels of the merged image in **(A)** are pseudo-colored with the Kindlmann color map for range visualization of the fluorescent signal intensity. Fluorescent signal for actin and FMNL3 colocalize at the site of *N*.*g* invasion (PCC = 0.922) **(B)**, whereas actin and FMN1 do not (PCC = 0.066). Fluorescent signal intensity profiles are shown in **(B and D)** for line 1 drawn along the invading colony and line 2 drawn across another area of the cell. **(E-G)** Plasma membrane region proximal to surface-associated gonococcal colonies **(E)** or aggregates of heat-killed gonococci **(F)** did not accumulate FMNL3 and actin microfilaments. **(G)** Quantitative analysis of FMNL3 accumulation at plasma membrane sites in contact with live gonococcal colonies or with aggregates of heat-killed gonococci. Means from two biological replicates ± SD. The statistical significance was calculated using the unpaired T-test. **(A-G)** Representative experiments from two **(F-G)** or at least three **(A-E)** biological replicates are included.

Because FMNL3 is required for gonococcal uptake, presumably by mediating actin polymerization at the sites of invasion, we investigated whether FMNL3 is localized there. Antibodies against FMNL3 did not work well for immunofluorescence microscopy; thus, we ectopically expressed FMNL3-V5 in U937 MFs. FMNL3-V5 was preferentially recruited at the sites of gonococcal entry (Fig 9A) where the formin co-localized extensively with the actin filament network surrounding the invading colony (Fig 9B). In contrast, FMN1-V5, another formins family member did not co-localize with the actin filaments at the site of *N*.*g* colony internalization (Fig 9C and 9D). Furthermore, surface-associated colonies that did not trigger actin polymerization also did not recruit FMNL3-V5 (Fig 9E) indicating that the two processes coincide. Importantly, neither actin nor FMNL3 were recruited by surface-associated aggregates of heat-killed gonococci (Fig 9F and 9G) indicating that the FMNL3-mediated actin polymerization leading to colony internalization is likely triggered directly or indirectly by the invading bacteria.

To ascertain the biological relevance of the FMNL3-mediated gonococcal uptake we investigated whether invasion of human macrophages protects gonococci from extracellular microbicidal activities. To this end, gonococci were allowed to invade U937 MFs for 6 hours at which point gentamicin was added for additional 2 hours to kill the non-internalized bacteria. In this assay, when invasion was blocked with cytochalasin D the number of protected bacteria decreased by ~90% (S5E Fig), moreover inactivation of *FMNL3* also phenocopied cytochalasin D treatment, albeit to a lesser extent (S5F Fig). Thus, the FMNL3-mediated gonococcal internalization by human macrophages is beneficial for the bacteria in this context.

### CEACAM1 is dispensable for gonococcal colony invasion of human macrophages

CEACAM1 is a well-established receptor for *N*.*g* colonization of several cell types including epithelial cells and neutrophils that can bind certain Opa family of gonococcal adhesins [42–44,82]. In neutrophil infections, Opa proteins binding to either CEACAM1 or CEACAM3 facilitates internalization, where engagement of CEACAM3 traffics gonococci to a degradative compartment and stimulates inflammatory cytokine secretion [46,47,83]. Because we could detect expression of CEACAM1 but not CEACAM3 and CEACAM5 in U937 MFs by qPCR (S8A Fig), we sought to determine if CEACAM1 acts upstream of FMNL3 to mediate gonococcal colony invasion. To this end, CEACAM1 KO U937 MFs were generated via genome editing (S8B Fig). Loss of CEACAM1 did not impact *Lp ΔdotA* phagocytosis nor gonococcal colony internalization by U937 MFs (S8C Fig) demonstrating that CEACAM1 is dispensable for invasion and intracellular colonization of human macrophages by *N*.*g*.

## Discussion

**Fig 10:**
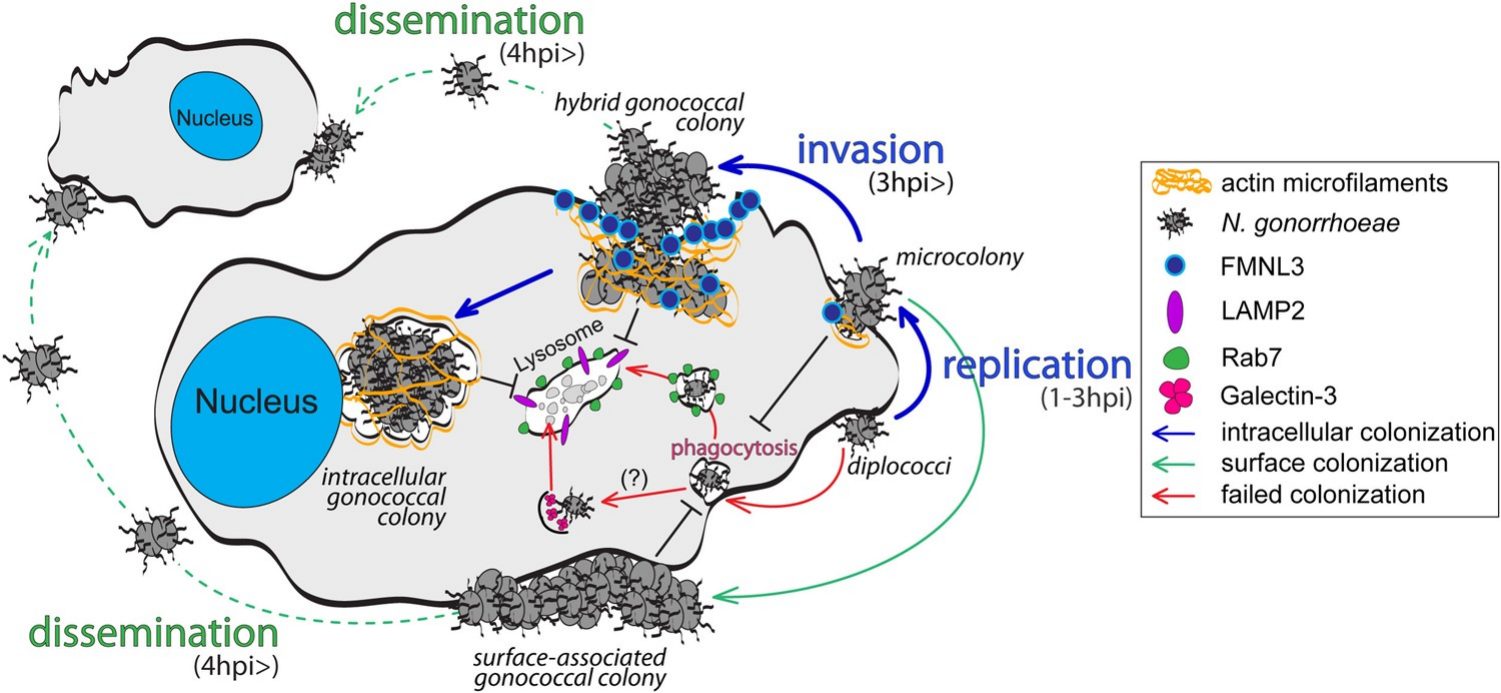
Model of macrophage colonization and invasion by gonococcal colonies. The diagram illustrates the proposed model of the three distinct stages of colonization of MFs by N.g and identifies several regulatory factors involved in the process (see “Discussion” for details).

Tissue-resident macrophages in the genitourinary tract, among other innate immune cells, likely encounter gonococci that have invaded the submucosa [2]. Here, we show that *N*.*g* colonizes human macrophages by establishing two distinct cellular niches, a surface-associated and an intracellular one, which can independently support bacterial replication. Our data indicate that macrophage invasion and establishment of an intracellular gonococcal colony is carried out by a surface-associated bacterial colony. This novel colonization paradigm is unlike other intracellular bacterial pathogens which invade host cells as single organisms. The model we propose in Fig 10 defines three distinct stages of colonization and identifies several regulatory factors. In the first stage (within 2hrs of contact), successful colonizers adhered to the macrophage surface, evaded phagocytosis and divided to produce microcolonies. Surface persistence was achieved by gonococcal colonizers via phagocytosis interference in a manner that allowed the uptake of other opsonized and non-opsonized cargo by the colonized macrophages. We propose that early evasion of phagocytosis is one critical step in macrophage colonization because gonococci that failed to block phagocytosis did not give rise to intracellular colonies but instead were transported to lysosomes and destroyed. After surface microcolonies are established (2–6 hpi), the colonization process branches in the second stage where majority of microcolonies begin to invade the host cell and the rest remain surface-associated. The sizes of invading colonies at any given time varied indicating that invasion is likely initiated somewhat stochastically. The appearance of intracellular colonies that reside within membrane-bound organelles marks the third stage of colonization. Within 24hrs, a significant percentage of gonococcal colonies were intracellular and some contained hundreds of bacteria.

Collectively, several lines of evidence demonstrate that intracellular colonies originated from surface microcolonies that invade the host cell via an atypical actin-dependent mechanism rather than phagocytosis of individual diplococci: (i) intracellular colonization was independent of several key canonical phagocytosis regulators—Rho GTPases, PI3K, Dynamin, Arp2/3; (ii) the Arp2/3 complex, a primary F-actin nucleator within the expanding phagocytic cup during cargo engulfment, was dispensable for gonococcal colony internalization; (iii) the formin FMNL3 was required for *N*.*g* invasion but was dispensable for phagocytosis in general; (iv) deletion of the phagocytosis regulatoring formins either increased (FHOD1) or did not affect gonococcal colony invasion (FMNL2, DIAPH2 and DAAM1); (v) internalized diplococci trafficked to the endolysosomal compartment, whereas invading colonies excluded EEA1, Rab7 and LAMP2; (vi) disruption of actin polymerization as late as 4hpi significantly reduced intracellular colonization even though a large number of diplococci were internalized within minutes.

All four different clinically-derived gonococcal strains we tested colonized U937 MFs and primary human MDMs similarly and produced surface-associated colonies as well as intracellular colonies decorated with actin filaments. Thus, gonococcal colonization of U937 MFs in many aspects recapitulates the colonization of the human primary monocyte-derived macrophages.

Gonococci invasion required FMNL3 but not its close homologs FMNL1 and FMNL2. FMNLs are members of the formins family of actin nucleators, which can assemble and bundle linear actin microfilaments [84]. FMNL3 localizes at filopodial tips and is one of six formins shown to promote filopodial protrusions at the plasma membrane [85]. In U937 MFs, loss of FMNL2, DIAPH2, FHOD1 and DAAM1 but not FMNL3 reduced non-specific phagocytosis (Fig 8). FMNL3 deletion reduced gonococcal colony invasion but the phenotype was less pronounced as compared to SMIFH2 treatment indicating that uptake might be regulated by other formins as well. However, recently discovered off-target effect of SMIFH2 (Formin^INH^) on Myosin II [86] might provide an alternative explanation for these results, i.e. the combined inhibition of FMNL3 and myosin II by SMIFH2 enhances the phenotype. Gonococcal invasion is significantly less sensitive to myosin II inhibition as compared to non-specific phagocytosis indicating that extensive disruption of the actomyosin network is needed to achieve a block in gonococcal internalization. Clearly, the potential role of myosin motors in gonococcal invasion warrants further scrutiny.

FMNL3 and F-actin also accumulated specifically to plasma membrane regions where gonococcal colonies are internalized, which did not happen when aggregates of heat killed gonococci adhered to macrophages (Fig 9), indicating that gonococci might directly or indirectly trigger FMNL3 activation. Formins typically fold in an auto-inhibited state where a C-terminal Diaphanous autoregulatory domain (DAD) engages a N-terminal FH3 domain [87]. Autoinhibition is relieved upon disruption of the DAD-FH3 interaction via higher-affinity binding to an alternative binding partner [88–91] or via post-translational modifications of the DAD region [92]. In this manner, formins function is tightly regulated at sites where actin polymerization is needed. When GTP-bound, members of the Rho family of small GTPases can bind formins and disrupt the autoinhibory state resulting in actin polymerization in a spatially controlled manner [90,93]. However, Rac1/2/3, Cdc42 as well as the Rho effector kinases LIMK1/2 and ROCK1/2 were dispensable for gonococcal colony uptake by macrophages; thus, further works is needed to determine the upstream events leading to FMNL3 activation. Several bacterial pathogens encode secretion systems effectors that can trigger actin polymerization for invasion [94,95]. For example, the *Salmonella* encoded type III secretion system effector SopE is a GEF for Cdc42 and Rac1 [96]. On the other hand, gonococci have not been demonstrated to deliver bacterial protein into the host cytosol [97,98]; thus, we speculate that FMNL3 activation is more likely mediated by engagement of a macrophage cell surface receptor. Both gonococci and meningococci can form epicellular colonies on epithelial cells, in which the base of the colony is partially embedded in actin-rich plasma membrane ruffles known as actin plaques [57]. Actin plaques cluster several surface receptors to recruit Ezrin—an adaptor protein that cross-links cell surface receptors with the cortical actin network [52–55]. Although on macrophages gonococcal surface colonies did not produce actin plaques nor recruit Ezrin (S2B and S6G Figs), analogous mechanisms, in principle, might lead to FMNL3 activation.

Gonococci uptake by neutrophils is mediated by the engagement of Opa adhesins with the human CEACAM1/CEACAM3 surface receptors and is strictly species specific [99,100]. We found that U937 MFs did not express CEACAM3 and that CEACAM1 is dispensable for macrophage entry. Similar to macrophage infections, gonococci were found to be both intracellular and surface-associated in neutrophil infections [27–29,60–62]. Thus, the entry and actin subversion mechanisms utilized by gonococci for macrophages, neutrophils and epithelial cells colonization appear distinct but share some common principles.

During invasion the gonococcal colony stretches from the surface of the macrophage and into a plasma membrane invagination that cradles a fraction of the colony. In this setting, surface gonococci acted as a plug to seal the opening of the membrane invagination thus producing a quasi-intracellular membranous compartment that is enveloped in polymerized actin microfilaments. The sharp discrete delineation observed between the protected (intracellular) and the unprotected (surface exposed) fractions of the invading colony in our inside/out immunofluorescence studies demonstrate that gonococci and the plasma membrane are tightly opposed each other at the transition zone, which rendered gonococci within the membrane invagination protected from antibodies in the extracellular milieu. Indeed, actin microfilaments were found to bracket bacteria at the neck of the plasma membrane invagination occupied by hybrid colonies. How formins produce these plasma membrane invaginations is to be determined. Nevertheless, to our knowledge this is the first example of bacterial pathogen entry mechanism solely based on linear actin microfilaments that does not require a functional Arp2/3 complex. Entry mechanisms by other intracellular bacteria require both linear and branched actin microfilaments (similar to lamellipodia formation) [63,64]. One examples is the uptake of *Borrelia burgdorferi* by primary human macrophages via coiling phagocytosis, which is mediated by the formins FMNL1 and DIAPH1 as well as the Arp2/3 complex [101]. A phagocytosis-like process has been shown to facilitate *Listeria* internalization by endothelial cells via FMNL3 and FHOD1-dependent actin polymerization in a PI3K-dependent manner [102]. However, our data demonstrate that PI3K and FHOD1 are dispensable for gonococcal invasion of macrophages indicating distinct regulatory mechanisms.

Our studies demonstrated that in macrophage infections gonococci can replicate intracellularly within a membrane-bound organelle as well as within a plasma membrane associated colony. Gonococci have been reported to localize in the cytosol of epithelial cells [27,28] and macrophages [12]; thus, it has been speculated that bacterial replication takes place in the macrophage cytosol. Infrequently, we detected individual diplococci in the cytosol of infected macrophages. Rupture of bacterium-containing phagosome can deposit vacuolar pathogens in the host cytosol, which are subsequently cleared by autophagy [103,104]. It is possible that cytosolic escape is a byproduct of a defect in entry, which would explain why single bacteria, but not gonococcal colonies localized to the cytosol. Potentially, macrophages might be more efficient at autophagic clearance of cytosolic bacteria as compared to epithelial cells, thus preventing gonococcal replication in the cytosol. Collectively, our data highlights that gonococci likely have evolved to establish multiple distinct cellular niches simultaneously. This phenomenon has obvious implications in immune evasion. A surface-associated niche protects from a number of cell autonomous defense responses targeting intracellular pathogens (such as lysosomal degradation, autophagy, and host cell death responses) [103–105], whereas an intracellular niche safeguards against humoral defense factors that target extracellular bacteria (such as complement, circulating antibodies and anti-microbial peptides) [106]. Perhaps adaptation to multiple topologically distinct niches could be an elegant evolutionary solution counteracting multiple inherently distinct host defense responses albeit at the cost of protecting only a fraction of the bacterial population.

## Material and methods

### Bacterial strains and culture conditions

*Neisseria gonorrhoeae* isolates FA1090, F62, MS11 and F19 were used in this study. All *Neisseria* strains were a kind gift from Dr. Hank Seifert (Northwestern University). Bacteria were cultured on gonococcal medium base (GCB) agar (Criterion) with Kellog’s supplements [107] at 37°C and 5% CO_2_ for approximately 16 hrs. For infections, 4 to 6 piliated Opa+ colonies as determined by colony morphology were pooled, streaked on GCB agar (1x1cm patch) and grown for 16hrs. Patches were collected, resuspended in PBSG (PBS supplemented with 7.5mM Glucose, 0.9 mM CaCl_2_, and 0.7 mM MgCl_2_) and the number of bacteria were determined by OD_550_ measurements. Inoculums for infections were prepared in PBSG and the number of bacteria were confirmed by plating dilutions from the inoculum on GCB agar. For experiments with heat killed bacteria, the inoculum was split, and one set was heat-killed at 70°C for 30min. Loss viability was confirmed by plating the bacteria on GCB agar.

*Legionella pneumophila* serogroup 1 strain LP01 with a clean deletion of the *dotA* gene was used in this study [74]. The *Legionella* strain was grown on charcoal yeast extract (CYE) plates [1% yeast extract, 1%N-(2-acetamido)-2-aminoethanesulphonic acid (ACES; pH 6.9), 3.3 mM l-cysteine, 0.33 mM Fe(NO3)3, 1.5% bacto-agar, 0.2% activated charcoal] [108]. For the macrophage phagocytosis studies, *Legionella* were harvested from CYE plates after growth for 2 days at 37°C, resuspended in H_2_O and bacterial number was determined by OD_550_ measurement.

### Calculation of bacterial growth rate

The mean generational times for the different gonococcal strains grown in axenic GCBL media or in the presence of U937 MFs cultured in PBSG were calculated based on viable CFU counts. CFUs were recovered at 1, 3, 5 and 8 hour and plotted to determine the log phase of the growth curve. The Δ CFUs between the 3-hour and the 8-hour time points were selected because they fall within the logarithmic growth phase in both types of experiments. For each time point, the average CFUs counts from four technical replicates were used for the growth rate calculation. First, the growth rate constant **μ** was calculated from the equation **μ** = ((*Log*_10_ CFU_8hrs_—*Log*_10_ CFU_3hrs_)*2.303) / (T_8hr_−T_3hr_). Next, the mean generation time **g** was calculated from **μ** = 0.693/**g**.

### Cells and culture conditions and viability assay

The human monocytic cell line U937 (ATCC CRL-1593.2) was obtained from ATCC and primary human peripheral monocytic cells (PBMCs) from two different donors were purchased from Precision for Medicine. U937 cells were cultured in RPMI 1640 media (VWR) supplemented with 10% Fetal Bovine Serum (FBS) at 37°C and 5% CO_2_. For differentiation into mature adherent macrophages, U937 monocytes were seeded at the desired density, cultured with 10 ng/ml phorbol 12-myristate 13-acetate (PMA) for 24hrs followed by 48hrs incubation with RPMI media (+10% FBS) in the absence of PMA. PBMCs were differentiated into mature macrophages with human recombinant macrophage colony stimulating factor (BioVision) in RPMI 1640 media supplemented with 20% human AB serum (Corning) for 7 days.

For viability studies, U937 cells were directly differentiated in white wall 96-well plates and treated with different doses of SMIFH2 (from 12.5μM to 100μM) or vehicle control (DMSO) for 8 hours. Each treatment condition was completed in six technical replicates. Cell viability was assessed using the CellTiter Glo 2.0 assay (Promega), which measures ATPs production in live cells. The assay was performed according to the manufacturer’s instructions. Maximum cytotoxicity (lowest luminescence signal) was determined by acute hypotonic lysis of U937 MFs. In a control treatment, cytotoxicity was also induced by acute (10 min) treatment with 0.02% Triton X-100 prior to measurement of cell viability.

### Antibodies and Inhibitors

The purified chicken IgY anti-*Neisseria gonorrhoeae* antibody was raised against formalin-killed FA1090 gonococci by Cocalico Biologicals and it cross-reacts with the MS-11, FA19 and F62 strains. The rabbit anti-*N*.*g* polyclonal antibody (cat# 20-NR08) was purchased from Fitzgerald Industries. Rabbit monoclonal antibodies against the following antigens were purchased from Cell Signaling—Rab5 (C8B1), Rab7 (D95F2), EEA1 (C45B10), GM130 (D6B1), Ezrin (#3145), LC3A/B (D3U4C), V5-Tag (E9H80), CEACAM1 (D3R80) and ACTR2 (#3128S). Mouse antibodies against the following antigens were purchased from Santa Cruz–Actin (sc8432), FMNL1 (sc390466), FMNL2 (sc390298), FHOD1 (sc365473), DAAM1 (sc100942). Mouse anti-Cas9 (7A9-3A3) was purchased from Cell Signaling. Anti-FMNL3 (ab57963) and anti-Galectin 3 (ab2785) mouse monoclonal antibodies were purchased from Abcam. Anti-human LAMP-2 (H4B4) was purchased from BioLegend. Anti-DIAPH2 (A300-079A-T) was purchased from Bethyl Labs. Highly cross-absorbed Alexa fluorophore conjugated secondary antibodies were purchased from Life Technologies.

Inhibitors used in this study were purchased from: (1) Cayman Chemicals—cytochalasin D (5μM), CK869 (10μM), Dynasore (30μM), GSK269962 (1μM), Nocodazole (3μM), ML-141 (5μM), EHT 1864 (5μM), BMS-5 (1μM) and (-) blebbistatin (5μM); (2) Cell Signaling—LY294002 (10μM); (3) Abcam—SMIFH2 (12.5μM to 100μM).

### Bacterial survival assays

Macrophages differentiated in 24-well plates at 2x10^5^ cells/well were infected with an inoculum of gonococci grown as a patch from 4–6 piliated colonies on GCB agar for 16hrs. The patch was collected in pre-warmed PBSG medium, the bacterial number was determined by OD_550_ densitometry and the MOI of the inoculum was at 1 bacterium per 10 macrophages. Bacteria were added to macrophages-containing wells in PBSG or empty wells on the same plate. The plate was centrifuged for 5min at 1000 rpm to bring the bacteria in contact with the macrophages. The T_0_ CFU counts were determined by plating a dilution of the inoculum on GCB agar. As indicated in each experiment, at different times post infection the number of the gonococci in the supernatant and in lysed cells (0.05% Tween-20 for 5 min; 37C) were determined by serial dilutions plated on GCB agar for CFU counts. Three technical replicates were done for each condition in each experiment.

### Contact-dependent bacterial replication assay

Macrophages differentiated in 6-well plates at 1x10^6^ cells/well were infected with an inoculum of gonococci grown on GCB agar for 16hrs as a patch started from 4 to 6 piliated colonies. The patch was collected in pre-warmed PBSG medium, the bacterial number was determined by OD_550_ densitometry and the MOI of the inoculum was at 2 bacteria per macrophage. The inoculum was prepared with PBSG and the infection was carried out in PBSG. Transwell inserts with a 0.4μm pore size bottom membranes (Corning) were placed in each well. The inoculum was added either within the transwell insert to measure contact independent bacterial replication or in the well to measure contact dependent replication. To establish a baseline of gonococcal replication in PBSG the inoculum was also added to wells that did not contain macrophages. The plate was centrifuged for 5min at 1000 rpm to bring the bacteria in contact with the macrophages. The T_0_ CFU counts were determined by plating a dilution of the inoculum on GCB agar. The number of the gonococci in the supernatant and in cells was determined by CFU counts as described. To confirm the transwell membrane integrity in the contact independent growth conditions, macrophages were lysed with detergent, serial dilutions were plated on GCB agar, and as expected viable CFU were not recovered. Three technical replicates were performed for each condition in each experiment.

### Immunofluorescence microscopy of infected macrophages

For infections, U937 monocytes were PMA-differentiated directly on cover slips in 24-well plates whereas hMDMs were seed on cover slips in media (RPMI + 10% FBS) for 20hrs prior to infection. The seeding density for all cell types was kept at 2x10^5^ cells/well. The MOIs used for each experiment and the duration of infection are indicated in the Figure legends. All infections were carried out in PBSG. We did not observe increase in macrophage cell death even after prolonged (> 24hrs) culture with PBSG. After the inoculum addition, cell culture plates were spun down at 1,000 rpms for 5min to facilitate bacteria-macrophage contact.

For immunofluorescence microscopy analysis, coverslips were washed with PBS three times, fixed with 2% paraformaldehyde (PFA) for 60 min at ambient temperature, permeabilized with 0.1% Triton X-100 for 20min, and blocked with 2% goat serum in PBS for 60 min. Next, primary antibodies were added in PBS containing 0.2% goat serum either overnight at 4°C or for 90min at room temperature. The antibodies dilutions were 1:500 for the α-*N*.*g* antibodies and 1:200 for all other primary antibodies. Secondary antibodies were used at 1:500 dilution and Hoechst 33342 at 1:2000 for 90 min at room temperature. The high affinity actin probe Alexa Fluor-568 Phalloidin (ThermoFisher) was used at 1:2000 dilution and was added with the secondary antibodies. Coverslips were mounted with ProLong Gold antifade reagent (ThermoFisher) onto slides and examined by fluorescence microscopy.

The different bacterial sub-populations in the inside/out staining methodology were detected by differential immunolabeling before and after detergent permeabilization of host cell the plasma membrane. Briefly, extracellular gonococci were immunolabeled with rabbit α-*N*.*g* antibody (Fitzgerald Ind.) in 2% goat serum for 90 min after the infected cells were washed with PBS three times, fixed with 2% PFA for 60 min at ambient temperature. The antibody was immobilized on the samples with 2% PFA (60 min incubation). Next, all bacteria were labeled with chicken α-*N*.*g* IgY antibody (Cocalico Biologicals) for 90 min after the samples were permeabilized with 0.1% Triton X-100 for 20min and blocked with 2% goat serum in PBS for 60 min. In each micrograph, antibody inaccessible gonococci were single positive whereas the surface associated bacteria were double positive.

### Microscopy analyses of infected cells

Images were acquired with inverted wide-field microscope (Nikon Eclipse Ti) controlled by NES Elements v4.3 imaging software (Nikon) using a 60X/1.40 oil objective (Nikon Plan Apo λ), LED illumination (Lumencor) and CoolSNAP MYO CCD camera. Image acquisition and analysis was completed with NES Elements v4.3 imaging software. Only linear image corrections in brightness or contrast were completed. For all analyses, three-dimensional images of randomly selected fields were acquired and image acquisition parameters were kept constant for all cover slips from the same experiment. The Z depth acquisition was set based on the out-of-focus boundaries and the distance between individual Z-slices was kept at 0.3μm.

### Analysis of gonococcal invasion

U937 macrophages differentiated on cover slips were pre-treated with the following inhibitors for 30 min prior to infection: cytochalasin D (5μM), CK869 (10μM), SMIFH2 (25μM), GSK269962 (1μM), LY294002 (10μM), Nocodazole (3μM), ML-141 (5μM), EHT1864 (5μM), BMS-5 (1μM), Dynasore (30μM) and (-) blebbistatin (5μM).

The inhibitors remained for the duration of the infection. Macrophages were infected with *N*.*g* FA1090 (MOI = 2) for 8hrs or with *L*. *pneumophila ΔdotA* (MOI = 40) for 2hrs, after which the cells were extensively washed with PBS and fixed with 2% PFA. Samples were processed for inside/out immunofluorescence analysis (as detailed in the previous section) and the number of internalized bacteria were determined by microscopy analysis.

### Bacteria quantitation using 3D immunofluorescence microscopy

To calculate the number of gonococci within a colony or the number of bacteria populating each host cell in 3D micrographs of gonococci-colonized macrophages we utilized the methodology we previously developed to count bacterial cells from *Legionella*-infected macrophages [109]. Briefly, 3D images (Z-slice = 0.3μm) of infected cells were analyzed by setting a binary mask using bacterial fluorescence to define individual objects. Within each object, the number of bacteria varied from individual diplococci to hundreds of bacteria (in large colonies). The formula to calculate the number of bacteria within an object volumes (***X*** *= 0*.4093*V*+1*.*134*, **R**^**2**^ = 0.9037, **X**-number of gonococci, **V**-object volume [voxels]) was derived from volume measurements data of individual bacteria as well as bacterial colonies (n = 142) where bacteria can be unambiguously identified and counted manually. Cortical actin was used to delineate the boundaries of individual infected macrophages.

### Macrophage phagocytosis assays

U937 macrophages differentiated on cover slips were infected with *N*.*g* FA1090 (MOI = 2) for 7hrs. Next, phagocytic cargo of live bacteria (MOI = 40) or latex particles were added for 60 min after which the cells were extensively washed with PBS and fixed with PFA. To measure FcR-mediated phagocytosis, FITC-labeled rabbit IgGs conjugated to latex beads with 0.1μm mean particle size (Cayman Chemicals) were used as cargo. The *Legionella pneumophila* Δ*dotA* was used to measure phagocytosis of live bacteria because this mutant is avirulent, is taken up via phagocytosis, traffics through the endocytic compartment and is killed by lysosomal degradation [74]. To determine cargo internalization, an inside/out staining methodology was utilized as follows: (1) fixed with 2% PFA (60 min), washed and blocked with 2% goat serum in PBS; (2) incubated with rabbit anti-*N*.*g* antibody (Fitzgerald Ind.) in the presence (Δ*dotA* infections) or absence (FITC-IgG latex beads treatment) of rabbit anti-*Legionella* antibody (Cocalico) for 60min; (3) fixed with 2% PFA (60 min), washed and permeabilized with 0.1% Triton X-100 for 20min; (4) incubated with purified chicken anti-*N*.*g* IgY antibody (Cocalico) in the presence (Δ*dotA* infections) or absence (FITC-IgG latex beads treatment) of purified chicken anti-*Legionella* IgY antibody (Cocalico) for 60min; (5) washed extensively and incubated with highly cross-absorbed anti-rabbit Alexa568 (ThermoFisher), anti-chicken IgY FITC (Life Technologies), and Hoescht 33342 (Molecular Probes) for 60 min. Size and morphology were used as determinants to differentiate between *Neisseria* (diplococcus, ~1μm), *Legionella* (rod, ~1.5μm) and the latex beads (amorphous aggregates).

### Thin-section transmission electron microscopy

Differentiated U937 macrophages were infected with *N*.*g* FA1090 for 10hrs (MOI = 2) on cover slips. The cells were washed with PBS and fixed with 1.6% glutaraldehyde and 2.5% paraformaldehyde in 0.1M Cacodylate Buffer for 60 min at ambient temperature. The samples were processed and imaged at the LSU School of Veterinary Medicine Imaging Core facilities (https://www.lsu.edu/vetmed/cbs/facilities/microscopy_center/index.php) on a JEOL JEM-1011 transmission electron microscope.

### Quantitative Real-time PCR analysis

U937 macrophages seeded in 24-well plates (2X10^5^ cells/well) were infected with *N*.*g* FA1090 for 8hrs (MOI = 2). In parallel, samples from uninfected cell were also collected. Total RNA isolation and first-strand cDNA synthesis was performed using TaqMan Gene Expression Cells-To-Ct Kit (ThermoFisher) as recommended by the manufacturer. Amount of the different mRNAs were determined by quantitative real-time PCR using the Universal ProbeLibrary (Roche, Life Science) and LightCycler 480 Probes Master (Roche, Life Science). The primers detecting the different CEACAMs mRNAs and their splice variants were designed by the Universal ProbeLibrary Assay Design Center (Roche) and are listed in S1 Table. Thermal cycling was carried out using a LightCycler 96 instrument (Roche Diagnostics) under the following conditions: 95°C for 5 minutes and 45 cycles at 95°C for 10s and 60°C for 25s. Gene expression was normalized to GAPDH.

### Generation of U937 stable cell lines

Formins knockout U937 cell lines were generated using lentiCas9-Blast (Addgene plasmid 52962) and lentiGuide-Puro (Addgene plasmid 52963) as previously described [110]. Guide RNAs used in this study are listed in S2 Table.

FMNL3-V5 and FMN1-V5 were purchased from Arizona State University Biodesign Institute DNASU Plasmid Repository. FMNL3-V5 and FMN1-V5 were expressed in WT or FMNL3 KO U937 MFs using lentiviruses generated in 293E cells co-transfected with the packaging constructs psPAX2 (Addgene plasmid 12260) and pCMV-VSV-G (Addgene plasmid 8454).

U937 cell lines stably expressing red fluorescent protein membrane were generated using pDsRed-Monomer-Mem (Takara Bio) cloned into pLB vector from Addgene (Addgene plasmid 11619) as previously described [111]. The corresponding lentiviruses were generated in 293E cells co-transfected with the packaging constructs psPAX2 and pCMV-VSV-G as described.

### Statistical analysis

Calculations for statistical differences were completed by Student’s T-test or multiparametric ANOVA analysis as indicated using Prism v9 (GraphPad Software).

## Supporting information

S1 Fig Kinetics of U937 MFs infections with *N*.*g* FA1090. Micrographs showing representative fields of macrophages infected with *N*.*g* FA1090 (MOI = 0.1) over 22hrs. Gonococci were detected with polyclonal chicken anti-*Ng* IgY antibody. Hoechst labels the macrophage nucleus. The arrowhead points to a super-infected macrophage at 22 hpi which contains hundreds of gonococci. Representative images from at least three biological replicates are included.
(TIF)

S2 Fig Distinct actin assembly at gonococcal colonies on hMDMs and epithelial cells. **(A)** Representative micrograph showing an actin plaque underneath a surface-associated *N*.*g* FA1090 colony at 8hpi on HeLa229 cells. **(B)** Quantitative analysis of actin plaque formation underneath surface-associated colonies on HeLa229 cells and U937 MFs infected with *N*.*g* FA1090 for 8 hours. Means of technical triplicates ± SD are shown, ** p < 0.005 unpaired T-test. **(C)** Micrograph of human MDMs infected with FA1090 at 8hpi showing representative neighboring macrophages that harbor either a hybrid (arrowhead) or a surface-associated colony (open triangle). Honeycomb pattern shaped actin cages assemble around intracellular regions of the hybrid colony, but actin plaques are absent underneath the surface colony. Individual focal plains of a 2μm Z-stack are shown. The actin channel is shown individually in grayscale. (**D**) Micrograph (z-stack projection) of a hybrid colony on a hMDM infected with *N*.*g* FA1090 at 8hpi that is also shown in 3D in the supplementary movie 1. **(A, C-D)** Colony topologies were determined by inside/out 3D microscopy. **(A-D)** Representative experiments from two **(C-D)** or at least three **(A-B)** biological replicates are included.
(TIF)

S3 Fig Quantitative analysis of gonococcal uptake by U937 MFs infected with *N.g* FA1090. The localization for each bacterium was scored based on inside/out microscopy of infected cells as indicated. (A) Presence of gonococcal microcolonies (4 to 12 bacteria) at 2hpi on U937 MFs infected at MOI = 2 with live or heat-killed bacteria. (B) Percentage of live and heat-killed gonococci internalized by macrophages at 1hpi (MOI = 2). (C) Analysis of the topology of U937 MFs-associated gonococcal microcolonies is shown. (D) Representative micrographs of a hybrid microcolony on a U937 MF at 4hpi from inside/out stained sample is shown. Single plane and z-projection images are shown. (A-C) Means of technical triplicates ± SD, statistical analysis—unpaired T-test. At least 100 bacteria or microcolonies were scored for each condition. (A-D) Representative experiments of three biological replicates are included.
(TIF)

S4 Fig Opsonized and non-opsonized cargo uptake by gonococci colonized U937 MFs. **(A-C)** Representative micrographs of U937 MFs pulsed with opsonized IgG-coated latex beads (0.1 μm mean particle size) or non-opsonized live *L*.*p ΔdotA* bacteria (MOI = 40) for 60min. Z-stack projections of inside/out stained samples are shown. Arrowheads indicate internalized cargo, arrows indicate extracellular cargo and asterisks indicate surface-associated gonococci. (**A**) IgG-beads uptake by uninfected U937 macrophages. **(B-C)** Cargo uptake by U937 macrophages colonized with gonococci for 7hrs (*N*.*g* FA1090). **(D)** Quantitative analysis of cargo uptake for 60min by uninfected macrophages vs. macrophages colonized with *N*.*g* FA1090. Data shows the percentage of uninfected macrophages with internalized cargo as percentage of all uninfected cells vs. the percentage of surface colonized macrophages with internalized cargo as percentage of all surface colonized macrophages. 3D inside/out microscopy was used to determine gonococci and cargo subcellular localization. Size and morphology were used as determinants to differentiate between *Neisseria* (diplococcus, ~1μm), *Legionella* (rod, ~1.5μm) and the latex beads (amorphous aggregates). Means of technical replicates ± SD are shown, at least 50 cells were analyzed for each condition. **(A-D)** Representative experiments from two biological replicates are included.
(TIF)

S5 Fig Quantitative analysis of intracellular and surface-associated gonococcal replication. **(A-B)** Surface-associated replication of *N*.*g* FA1090 on U937 MFs pre-treated with 5μM cytochalasin D or with DMSO. **(A)** Representative micrographs of large surface associated colonies at 8hpi on U937 MFs pre-treated with 5μM cytochalasin D. **(B)** Gonococcal CFUs recovered from 8 hour infections (MOI = 0.1) in the presence/absence of 5μM cytochalasin D. **(C-D)** Quantitative analysis of gonococcal invasion **(C)** and size of gonococcal colonies **(D)** at 10 hpi in U937 MFs infections when cells were either treated with DMSO or cytochalasin D at 4 hpi. Inside/out microscopy was used to determine the colony topology and 3D object volume measurement (in voxels) was used to determine the size of each macrophage-associated *N*.*g* colony. Each data point represents an individual colony and “n” indicates the number of *N*.*g* colonies analyzed in each condition. Means ± SD are shown, unpaired T-test analysis. **(E-F)** Macrophage invasion by gonococci is protective. Decreased internalization of *N*.*g* by U937 MFs due to actin polymerization inhibition **(E)** or loss of FMNL3 **(F)** reduced the number of viable gonococci recovered at 8hpi after gentamicin treatment for 2h (50μg/ml). Means of technical triplicates ± SD are shown, unpaired T-test analysis. **(A-F)** Representative experiments from at least three biological replicates are included.
(TIF)

S6 Fig Cellular distribution of different host proteins in U937 MFs colonized by *N*.*g* FA1090 gonococci. **(A-E)** Single focal plane representative micrographs from 3D images showing *N*.*g* FA1090 gonococci in U937 MFs. **(A, D and E)** Micrographs of gonococcal colonies residing in cellular compartments devoid of **(A)** Rab7, **(D)** LAMP2 and **(E)** Rab5 are shown. **(B and C)** Micrographs of gonococci residing in **(B)** Rab7 positive, or **(C)** LAMP2 positive compartments are shown. **(F)** Micrographs showing LC3 recruitment to cytosolic gonococci. **(E)** Ezrin localization in U937 MF harboring a gonococcal colony. **(A-G)** Representative experiments from at three biological replicates are included.
(TIF)

S7 Fig SMIFH2 effects on U937 MFs viability. U937 MFs were treated with different doses of SMIFH2 or vehicle control for 8 hours as indicated, and cell viability was assessed using the CellTiter Glo 2.0 assay (Promega). Acute (10 min) treatment with 0.02% Triton X-100 was used to induce cytotoxicity. Means of six technical replicates ± SD are shown. A representative experiment from two biological replicates is included.
(TIF)

S8 Fig Gonococci invasion of human macrophages is independent of CEACAM1. **(A)** qPCR mRNA analysis for *ceacam1*, *ceacam3*, *ceacam5* and *ceacam6* was carried out on U937 MFs that were either infected with *N*.*g* FA1090 for 8hr (MOI = 2) or were left uninfected. Primer sets specific for different transcripts splice variants (*tv*) were designed and used when possible. Means of biological triplicates ± SD are shown. **(B)** Immunoblot shows the loss of CEACAM1 in the CEACAM1 KO U937 MFs. **(C)** Graph shows quantitative analysis of *N*.*g* colony and *Lp ΔdotA* internalization by the CEACAM1 KO U937 MFs using inside/out microscopy. The Internalization index for each condition was calculated by dividing the percentage of internalized objects from infections of the CEACAM1 KO U937 MFs cell line by the percentage of internalized objects from infections of the parental U937 MFs, which was then multiplied by 100. Values > 100% indicate increased object internalization by the KO cell line compared to WT cells, whereas values < 100% indicate decreased object internalization by the KO cell line. At least 100 objects for each condition were scored. Means from three biological replicates ± SD. The statistical significance of the differences between the internalization index from the KO cells and the parental cells for each condition were calculated using the unpaired T-test. **(A-C)** Representative experiments from at three biological replicates are included.
(TIF)

S1 Video Representative 3D micrograph of a hybrid gonococcal colony invading a human primary monocyte-derived macrophage. The movie is assembled from sequential single focal plane micrographs along the Z-axis which are separated by 0.3μm from each other. The video shows a macrophage infected with *N*.*g* FA1090 that is processed for inside/out microscopy. Purple arrow indicates the gonococcal colony. White arrows point to the surface exposed bacteria from the colony, which are false colored green.
(MP4)

S1 Table Primers and probes used for Q-PCR. Forward (F) and Reverse (R) primers and corresponding Universal Library Probes (Roche) used for Q-PCR analysis of human formins and *ceacam* genes expression are presented. When possible, individual transcription variants (tv) were analyzed.
(PDF)

S2 Table Guide RNAs used for U937 knockout (KO) lines. Guide RNAs used for formins, ACTR2 and CEACAM1 U937 KO lines are presented. The guide RNAs were cloned into lentiGuide-Puro (Addgene plasmid 52963).
(PDF)
