# Supplementary material for: Neisseria gonorrhoeae subverts formin-dependent actin polymerization to colonize human macrophages
Source: PLoS Pathog. 2021 Dec 28;17(12):e1010184. doi: 10.1371/journal.ppat.1010184 (PMC8746766; doi:10.1371/journal.ppat.1010184)
Supplement: S1 Table — Forward (F) and Reverse (R) primers and corresponding Universal Library Probes (Roche) used for Q-PCR analysis of human formins and ceacam genes expression are presented. When possible, individual transcription variants (tv) were analyzed. (PDF) [file ppat.1010184.s010.pdf]

S1 Table.

Name	Sequence (5’→ 3’)	Probe Number
F_CEACAM1_tv345	tacctgccacgccaataact	71
R_CEACAM1_tv3	tgggtaacatggtgagattctg	71
R_CEACAM1_tv45	aggccattttcttgtggtagag	71
F_CEACAM1_tv126	cccatcatgctgaacgtaaa	57
R_CEACAM1_tv126	agggccactactccaatcac	57
F_CEACAM3	cacagtagccctgactacagca	1
R_CEACAM3	gtctctgctgcctgctcttt	1
F_CEACAM5_ tv1	catagtcaagagcatcacagtctct	2
R_CEACAM5_tv1	tcatgatgccgacagtgg	2
F_CEACAM5_tv23	tcaagagcatcacagtctctgc	2
R_CEACAM5_tv23	atcatgatgccgacagtgg	2
F_CEACAM6	cggcatcacgattggagt	21
R_CEACAM6	gaaaatacaccagggctgcta	21
F_ DAAM1_tv12	ggagctacaagttggcctga	46
R_ DAAM1_tv12	tccttctctaaagccagcaga	46
F_ DAAM2_tv12	aagagctcccactgcaagac	60
R_ DAAM2_tv12	ctttagaggcctcacccaca	60
F_ DIAPH1_tv123	tctgcccagaccaagacttc	39
R_ DIAPH1_tv123	tttttcttttgcacagatttctttt	39
F_ DIAPH2_tv1256	ccaccaaacgtgagatggtt	59
R_ DIAPH2_tv1256	tttttcagcccaccagattt	59
F_ DIAPH3_tv1235	ttcatgcaagcaataaaggaga	6
R_ DIAPH3_tv1235	ttctttagctattctgacacgttttt	6
F_ FMN1_tv13	gtacccaaagccgacttgc	1
R_ FMN1_tv13	cgatgacctatccccttgc	1
F_ FMN2	aaaccagccacgaacactct	6
R_ FMN2	ggtggagatgggatgttacag	6

F_ FMNL1	ctttgcccagtgctctgtc	1
R_ FMNL1	tggacccttgctgaggtct	1
F_ FMNL2	cacaacgtgcctttgaagc	21
R_ FMNL2	ggaggtaggttcatagcattcag	21
F_ FMNL3	tggcatataccacccatctct	30
R_ FMNL3	gttcaagggtcccctactcc	30
F_ INF2_tv12	gaggtctttgcctccctgtt	32
R_ INF2_tv12	gacaggagctgggcagac	32
F_ FHOD1_tv12	gctccctcttctcactgaagc	79
R_ FHOD1_tv12	acaaattcaggcaccaggtc	79
F_ FHOD3_tv123	caagacatggatttcactgacc	52
R_ FHOD3_tv123	gacccaggtccacatctagg	52
F_ GRID2IP	ctgctgacctatgaggagca	2
R_ GRID2IP	cgtgtagcccaggaagca	2
