# Supplementary material for: Neisseria gonorrhoeae subverts formin-dependent actin polymerization to colonize human macrophages
Source: PLoS Pathog. 2021 Dec 28;17(12):e1010184. doi: 10.1371/journal.ppat.1010184 (PMC8746766; doi:10.1371/journal.ppat.1010184)
Supplement: S2 Table — Guide RNAs used for formins, ACTR2 and CEACAM1 U937 KO lines are presented. The guide RNAs were cloned into lentiGuide-Puro (Addgene plasmid 52963). (PDF) [file ppat.1010184.s011.pdf]

S2 Table.

GENE NAME	LIBRARY NUMBER	SEQUENCE
FMNL1	HGLibA_17692	GAGGGCCAACTCGCCCACCA
FMNL1	HGLibA_17693	GTGCTCCAGCACAGCGTTCT
FMNL1	HGLibA_17694	GCAGGGAGCGCACGTCCGCC
FMNL2	HGLibA_17695	ATCATACTGCCGCAGTAACC
FMNL2	HGLibA_17696	CTTTCTGCAGGAACGATTCC
FMNL2	HGLibA_17697	TCTTTGAGAACTAACCACAT
FMNL3	HGLibA_17698	CGGTGGAGGACATGAACTTC
FMNL3	HGLibA_17699	CAGAGCCATCATGAACTATC
FMNL3	HGLibA_17700	CCCAGGTATAGCACTCTCCC
DIAPH2	HGLibA_13219	GAACCGGGCCGCCAATGAAG
DIAPH2	HGLibA_13220	CCGCGCTCCGCTTGTTGCTC
DIAPH2	HGLibA_13221	CTTTAACCAGCAATCCGGTC
DAAM1	HGLibA_12258	ATCAACAATACCTCGATAGA
DAAM1	HGLibA_12259	CTCACCGGCTCATCTCAAAA
DAAM1	HGLibA_12260	CGTGTTTATGTTCCTCCAAT
FHOD1	HGLibA_17491	CTCTCCTCAGTCCCGCTTGG
FHOD1	HGLibA_17492	TACCAGAGCTACATCCTTAG
FHOD1	HGLibA_17493	GACCTCTAAGGATGTAGCTC
ACTR2	HGLibA_00655	TATAACTAGATATCTTATCA
ACTR2	HGLibA_00656	TCATTCCAGTTTGTGAAGTG
ACTR2	HGLibA_00657	CACATTTGCCCAGTATATGA
CEACAM1	HGLibA_08922	CACGCCAATAACTCAGTCAC
CEACAM1	HGLibA_08923	CATGCCATTCAATGTTGCAG
CEACAM1	HGLibA_08924	AGAGCTCTTGTGTGCTTTGC
MAP1LC3B	HGLibA_28123	TTCAAGCAGCGCCGCACCTT
MAP1LC3B	HGLibA_28124	GTGAGCTCATCAAGATAATT
MAP1LC3B	HGLibA_28125	GTACATGGTCTATGCCTCCC
